# Synergistic Hydrogen‐Bonding and Covalent Crosslinking in Polybenzimidazole Membranes for Wide‐Temperature Anhydrous Fuel Cells

**DOI:** 10.1002/advs.202522161

**Published:** 2026-02-24

**Authors:** Junming Dai, Jianming Zhong, Jinpeng Luo, Yuxing Song, Fan Hu, Young Moo Lee, Yongbing Zhuang

**Affiliations:** ^1^ State Key Laboratory of Biopharmaceutical Preparation and Delivery Institute of Process Engineering Chinese Academy of Sciences Beijing China; ^2^ School of Chemical Engineering University of Chinese Academy of Sciences Beijing China; ^3^ Department of Energy Engineering College of Engineering Hanyang University Seoul Republic of Korea

**Keywords:** dual‐crosslinking network, membrane electrode assembly, phosphoric acid doping, polybenzimidazole, wide‐temperature‐range proton exchange membrane

## Abstract

Expanding the operational temperature range and reducing the humidity dependence of proton exchange membrane fuel cells (PEMFCs) remain critical challenges. To address these issues, we developed a dual‐network architecture that integrates a thermally reinforced hydrogen‐bonding matrix with an amine‐anhydride covalent crosslinking framework within Tröger's Base (TB)‐functionalized polybenzimidazole membranes. The covalently crosslinked TBAm‐PBI‐TB membrane with dual‐network architecture exhibited a high phosphoric acid uptake of 469.5% with negligible leaching and achieved proton conductivities of 255.5 mS cm^−^^1^ at 90°C and 20% relative humidity (RH) and 264.7 mS cm^−^^1^ at 160°C under anhydrous conditions. It also demonstrated excellent oxidative and mechanical stability. A membrane electrode assembly (MEA) based on the TBAm‐PBI‐TB membrane delivered peak power densities ranging from 108.6 to 446.2 mW cm^−^^2^ between 30 and 160°C under anhydrous H_2_/air conditions. This maximum power density exceeds that of a Nafion 211‐based MEA, which reached 367.8 mW cm^−^^2^ at 30°C under 40%–50% RH. The MEA also showed outstanding operational durability. This work presents a strategy for developing wide‐temperature proton‐conducting membranes for anhydrous fuel cells.

## Graphical abstract

A dual‐network architecture combining a thermally reinforced hydrogen‐bonding matrix with an amine‐anhydride covalent framework was constructed in Tröger's Base (TB)‐functionalized polybenzimidazoles. This design yielded the TBAm‐PBI‐TB membrane with high acid uptake (469.5%), anhydrous conductivity (264.7 mS cm^−^^1^), and robust stability. The corresponding membrane electrode assembly delivered 108.6–446.2 mW cm^−^^2^ under anhydrous H_2_/air from 30°C to 160°C, surpassing a commercial Nafion 211 benchmark while maintaining excellent durability for humidifier‐free operation.

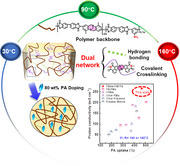

## 1 Introduction

Proton exchange membrane fuel cells (PEMFCs) are widely regarded as highly efficient energy conversion devices due to their pollution‐free operation and high specific power density [1, 2, 3, 4]. Proton exchange membranes (PEMs) capable of wide‐temperature operation have recently attracted growing interest due to their enhanced practicality in fuel cell applications [5, 6, 7]. Currently, Nafion membranes with hydrophobic fluorinated main chain and hydrophilic sulfonic acid side chain exhibit high proton conductivity under fully hydrated conditions [8], which are considered as the state‐of‐the‐art PEMs for low temperature operation (≤80°C) due to significant phase separation between the hydrophobic backbones and hydrophilic clusters [9, 10, 11, 12, 13]. However, Nafion suffers a dramatic loss in proton conductivity upon dehydration at temperatures exceeding 100°C [14, 15, 16].

High‐temperature proton exchange membrane fuel cells (HT‐PEMFCs) operate between 100 and 200°C without humidification, offering distinct advantages such as reduced catalyst poisoning, enhanced electrode kinetics, and simplified system design [17, 18]. Among HT‐PEM candidates, phosphoric acid (PA)‐doped polymers such as polybenzimidazole (PBI) are the most prominent materials [19, 20]. Nevertheless, few acid‐doped membranes exhibit adequate performance across a wide temperature range, primarily due to the lack of acid/water adsorption sites in their backbones, resulting in poor proton conductivity below 80°C [21, 22, 23]. Therefore, to promote the practical implementation of PA‐doped PEMs, extending the operational temperature range through effective materials design strategy is urgently needed [24, 25].

To this end, aromatic polymers with intrinsic microporosity have been explored to enhance PA uptake and water adsorption, enabling operation over a wide temperature range [5, 6, 26]. For instance, ultramicroporous membranes derived from Tröger's Base (TB) polymers achieve proton conductivity from ‐20°C to 200°C, benefiting from strong microporous siphoning and the high acid‐affinity of TB units [5]. Similarly, incorporating hydrophilic crown ether units into microporous copolyimides (co‐PIs) significantly boosts H_2_O/PA adsorption, supporting operation between 30°C and 160°C [27]. However, at higher PA concentrations, these membranes often suffer from excessive swelling and even dissolution due to limited PA tolerance [28], hindering their practical application.

Alternative strategies involve blending PBIs with hydrophobic materials to regulate H_2_O/PA adsorption and enhance proton conduction across wide temperature ranges. For instance, composite membranes incorporating three‐dimensional polyacrylamide hydrogels have been reported to operate effectively between 40°C and 180°C [6]. Nevertheless, residual monomers from the free‑radical polymerization process can compromise membrane integrity. Although blending phosphate‑functionalized carbon nanotubes with PBIs facilitates dual‑mode proton transport [24], these additives tend to aggregate during membrane casting, which limits both their loading and dispersion uniformity.

**FIGURE 1:**
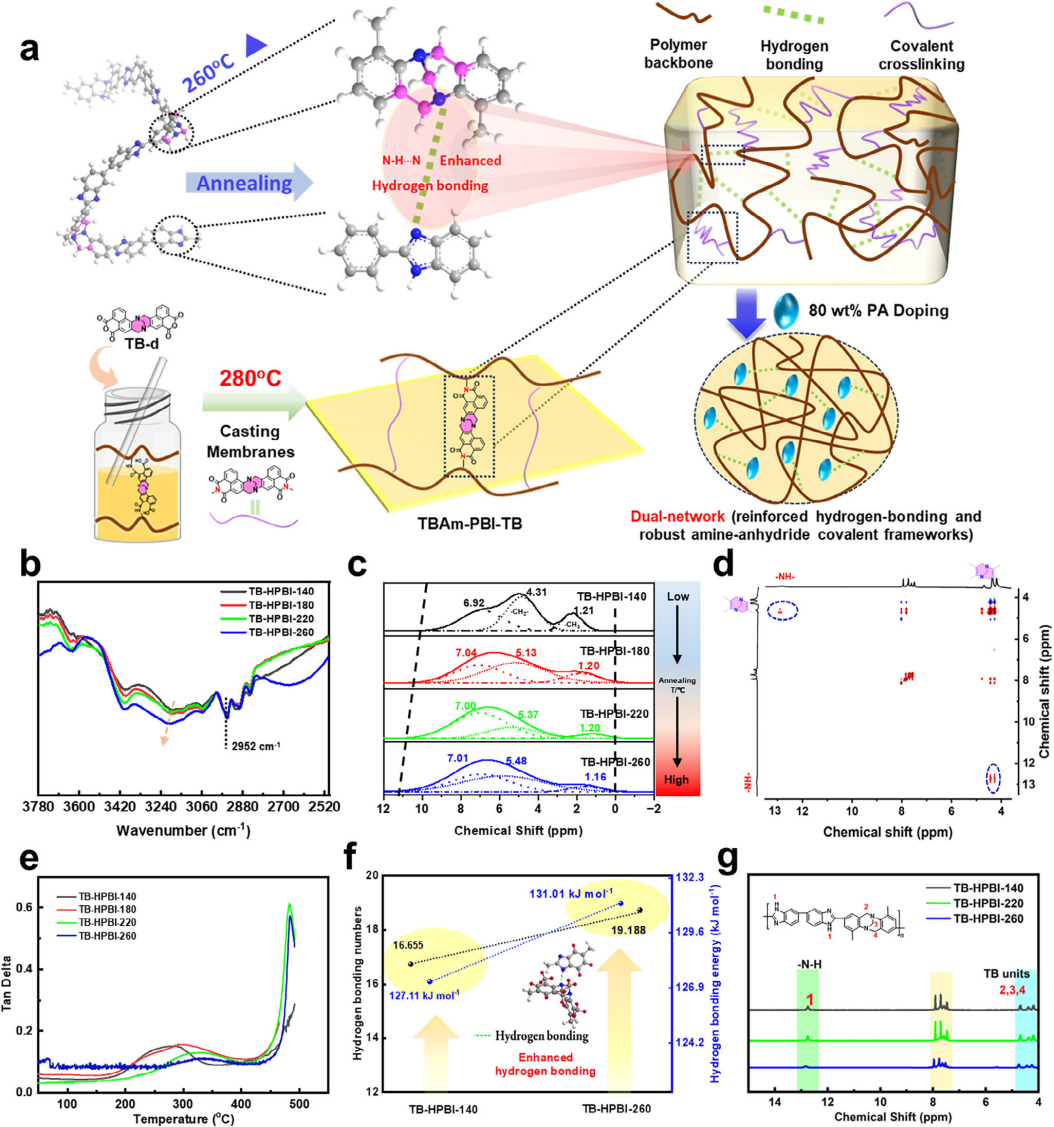
Strengthened hydrogen bonding within TB‐based PBI membranes. a) A dual‐network architecture integrating thermally reinforced hydrogen‐bonding matrices with robust amine‐anhydride covalent crosslinking frameworks. b) FT‐IR spectra of variously annealed TB‐HPBI membranes from 2500 to 3780 cm^−1^. The –N─H stretching band (3500–2500 cm^−^^1^) and an asymmetric C─H stretching band near 2952 cm^−^^1^ attributable to ─CH_3_ groups. c) Solid‐state ^1^H NMR of TB‐HPBI annealed at different temperatures. d) ^1^H‐^1^H 2D NOESY spectrum of TB‐HPBI‐220 in DMSO‑*d*_6_, indicating spatial proximity among proton environments. e) Temperature dependence of tan delta (tan*δ*) plots for the annealed membranes (DMA), illustrating their viscoelastic behavior. f) Simulated hydrogen bond counts and bonding energies for TB‐HPBI‐140 and TB‐HPBI‐260 membranes, indicating enhanced hydrogen bonding in the latter. g) ^1^H‐NMR spectra of TB‐HPBI‐140, TB‐HPBI‐220, and TB‐HPBI‐260 membranes. Due to the initial insolubility of TB‐HPBI‐260 in DMSO‑*d*_6_, it was first dissolved in 85 wt.% PA. The acid was subsequently removed by washing to enable dissolution in DMSO‑*d*_6_ for ^1^H‐NMR analysis.

TB‐based PBI membranes are promising candidates for HT‑PEMFCs due to their improved solubility and processability [29, 30, 31]; however, their proton conductivity remains limited below 80°C. To address this, we designed a dual‑network architecture within TB‑functionalized PBI membranes for anhydrous fuel cells operating over a broad temperature range. This architecture combines a thermally reinforced hydrogen‑bonding matrix with a robust amine‑anhydride covalently crosslinked framework (Figure 1a). A TB‑based PBI homopolymer (TB‐HPBI, Figure S1a) was synthesized. Strong hydrogen bonding between TB units and the imidazole N–H groups (denoted TBz) was confirmed and further enhanced by vacuum annealing at 140°C–260°C (Figure 1a). The effect of enhanced hydrogen bonding on membrane properties and fuel cell performance was systematically assessed. Furthermore, a TB‐based PBI copolymer functionalized with pendant amino groups (TBAm‐PBI, Figure S1b) was prepared. TBAm‐PBI was subsequently reacted with three dianhydrides‐4,4'‐(hexafluoroisopropylidene) diphthalic anhydride (6FDA), 1,4,5,8‐naphthalenetetracarboxylic dianhydride (NA), and a novel TB‐based dianhydride (TB‐d, Figure S2), followed by thermal imidization to produce covalently crosslinked membranes (Figure 1a; Figure S1c). The effects of the dual‑network structure on membrane properties, including PA retention, water uptake, and proton conductivity, as well as on fuel cell performance, were comprehensively discussed. This strategy significantly improved PA uptake and optimized water absorption, enabling high proton conductivity across a wide temperature window (30°C–160°C) under low‑humidity or anhydrous H_2_/air conditions.

## 2 Results and Discussion

### 2.1 Thermal Enhancement of Hydrogen Bonding

TB‐HPBI was synthesized following a reported procedure (Figure S1a) [31]. The obtained membrane was subsequently vacuum‐annealed at 140°C, 180°C, 220°C, and 260°C to produce the TB‐HPBI‐x series, where x represents the annealing temperature. As shown in Figure 1b, the Fourier Transform‐InfraRed (FT‐IR) spectra exhibited characteristic vibrations of the imidazole ring, notably the —N─H stretching band (3500–2500 cm^−^^1^) [32] and an asymmetric C─H stretching band near 2952 cm^−^^1^ attributable to ─CH_3_ groups. The N–H stretching band progressively broadened and intensified with increasing annealing temperature, suggesting a strengthening of interchain hydrogen bonding. This conclusion was further supported by solid‐state ^1^H Nuclear Magnetic Resonance (^1^H NMR) (Figure 1c), where higher annealing temperatures led to downfield broadening of aromatic proton signals and upfield broadening of methyl proton signals, consistent with enhanced interchain interactions such as hydrogen bonding and π–π stacking. A pronounced downfield shift of the TB methylene protons from 4.31 to 5.48 ppm suggested an increasingly electron‐withdrawing environment, directly corresponding to intensified hydrogen bonding involving TB units (denoted as TBz hydrogen bonding). Additional evidence came from the two‐dimensional Nuclear Overhauser Effect Spectroscopy (2D NOESY) of TB‐HPBI‐220 (Figure 1d), which showed cross‐peaks indicative of short‐range (<5 Å) interactions between –N─H protons and TB nitrogen atoms in dimethyl sulfoxide (DMSO)‐*d*_6_, confirming the formation of TBz hydrogen bonding [33, 34, 35].

To further confirm TBz hydrogen bonds, blended systems were prepared using benzimidazole (BI‑m) and a Tröger's base monomer (TB‑m) as model compounds (Figure S3). Keeping the concentration of BI‑m constant while increasing TB‑m resulted in a noticeable downfield shift of the imidazole N–H proton signal from 8.359 to 9.133 ppm. This shift reflects a decrease in electron density at the benzimidazole N–H proton due to stronger intermolecular hydrogen bonding. Correspondingly, the ─CH_2_─ proton signals of TB‑m also shifted downfield, which is attributed to the enhanced electron‐withdrawing effect of the tertiary amine nitrogen in the TB‐m. The observed shift results directly from the formation of intermolecular N–H(BI‑m)···N(TB‑m) TBz hydrogen bonds, thereby reducing the electron density surrounding the adjacent methylene groups. Solid‐state ^1^H NMR provided further evidence (Figure S4): whereas pure TB‑m exhibited a broad, hydrogen‐bonded benzimidazole N–H signal at 13.9 ppm, blending with BI‑m caused it to split into two resonances at 13.0 and 14.2 ppm, indicating rearrangement of the hydrogen‐bonding network. Concurrently, the TB methylene proton signals coalesced into a broad envelope around 3.6 ppm, further confirming the establishment of TBz hydrogen bonding between TB‑m and BI‐m.

Integrated evidence from dynamic mechanical analysis (DMA), differential scanning calorimetry (DSC), and molecular dynamics (MD) simulations demonstrate that thermal annealing strengthens hydrogen bonds. DMA (Figure 1e) showed *β*‐relaxation peaks between 200°C–400°C, associated with the rotation/vibration of benzimidazole groups [36, 37, 38]. The *β*‐relaxation temperature increased systematically from 273°C (TB‐HPBI‐140) to 347°C (TB‐HPBI‐260) induced by annealing. Correspondingly, the tan*δ* magnitude decreased at elevated annealing temperatures, indicating reduced energy dissipation. Concurrently, the storage modulus improved markedly with higher annealing temperature (Figure S5), further confirming the enhancement of hydrogen bonding network. Further evidence was provided by DSC analysis (Figure S6), which revealed a broad exothermic transition between 183°C and 262°C. This endothermic response corresponds to the initial formation and progressive strengthening of hydrogen bonds. MD simulations (Figure 1f; Figure S7) quantitatively validated this strengthening [34]: the hydrogen bond energy rose from 127.11 kJ mol^−1^ (TB‐HPBI‐140) to 131.01 kJ mol^−1^ (TB‐HPBI‐260), and the number of bonds increased from 16.655 to 19.188.

The reversibility of hydrogen bonding was confirmed through dissolution of TB‐HPBI‐260 in 85 wt.% PA, which disrupted the hydrogen‐bonding network. Following precipitation and purification, the recovered polymer readily redissolved in DMSO. The essentially identical ^1^H NMR spectra (Figure 1g) indicated an intact, non‐crosslinked structure. Moreover, elemental analysis revealed negligible compositional changes between TB‐HPBI‐180 and TB‐HPBI‐260 (Table S1), consistent with reversible hydrogen bonding and maintained structural integrity upon annealing.

### 2.2 Enhanced Performance Through Strengthened Hydrogen Bonding

**FIGURE 2:**
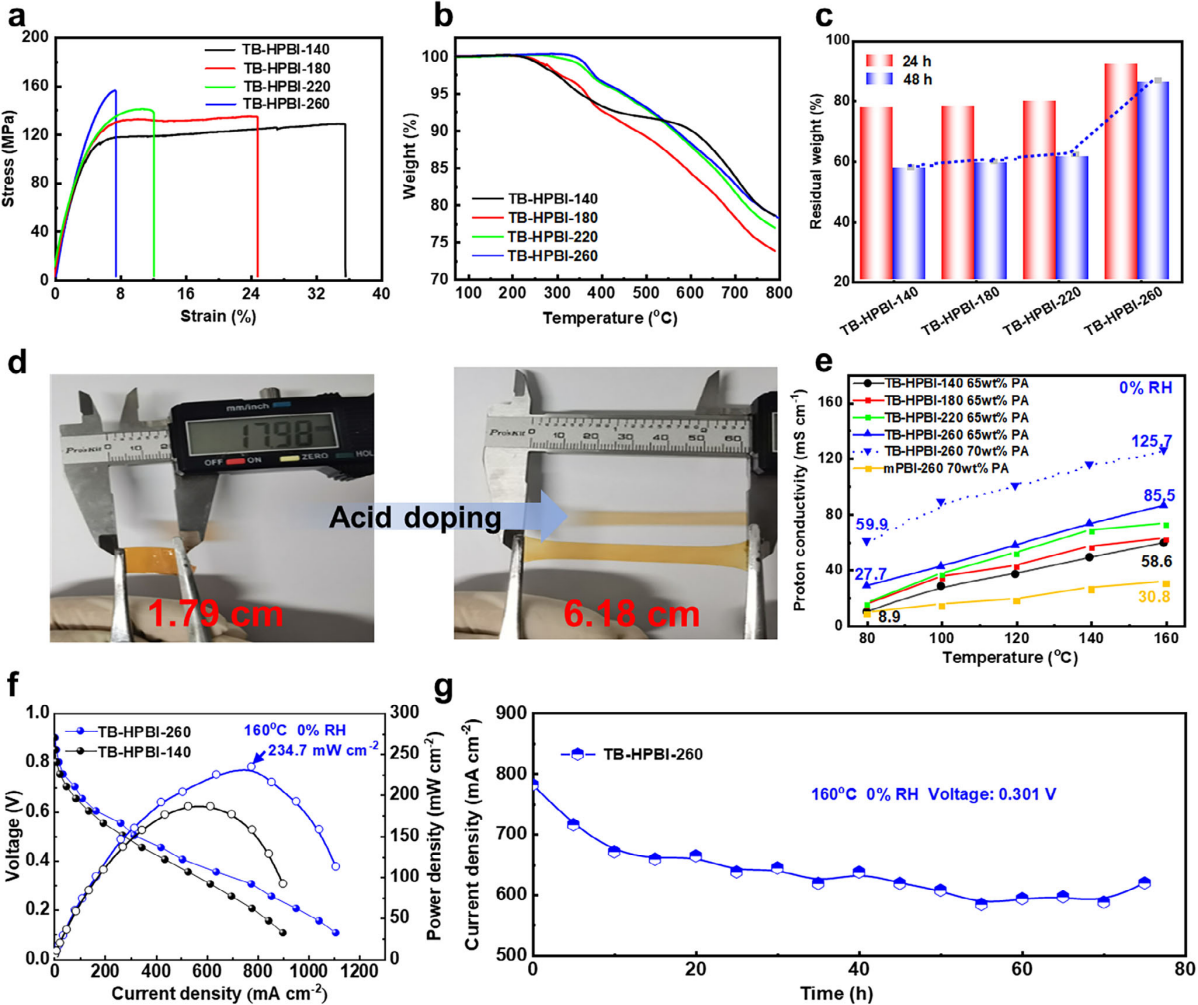
Effects of N─H⋯N hydrogen bonding on membrane properties and fuel cell performance. a) Stress–strain curves of the annealed TB‐HPBI membranes. b) Thermogravimetric analysis (TGA) curves of TB‐HPBI membranes annealed at different temperatures. c) Oxidative stability of annealed membranes assessed in Fenton's reagent at 80°C (3 wt.% H_2_O_2_, 4 ppm Fe^2+^). d) Toughness of the PA‐doped TB‐HPBI‐260 membrane. e) Proton conductivity of membranes doped with 65 wt.% or 70 wt.% PA in the 80°C–160°C range. f) Single‐cell performance (H_2_/air) of TB‐HPBI‐140 (65 wt.% PA) and TB‐HPBI‐260 (70 wt.% PA) membranes measured at 160°C. Anode/cathode catalyst loading: 1 mg_pt_ cm^−2^. g) Long‐term durability test of the TB‐HPBI‐260 membrane at 160°C under a constant voltage of 0.3 V.

Progressive reinforcement of hydrogen bonding improved both tensile strength and modulus [39]. Notably, TB‐HPBI‐260 exhibited substantially enhanced mechanical properties (Figure 2a), attaining a tensile strength of 157.2 MPa and a modulus of 4.0 GPa. These values represent increases of 23 % and 38 %, respectively, compared to TB‐HPBI‐140 (127.5 MPa, 2.90 GPa). Thermogravimetric analysis (TGA, Figure 2b) demonstrated improved thermal stability from enhanced hydrogen bonding. TB‐HPBI‐260 displayed initial decomposition at 338.6°C (vs. 226.6°C for TB‐HPBI‐140), along with elevated 1% and 5% weight‐loss temperatures. Oxidative stability tests in Fenton's reagent (80°C, 3 wt.% H_2_O_2_, 4 ppm Fe^2^^+^) revealed a marked performance difference among the membranes. Notably, TB‐HPBI‐260 retained 92.1% of its mass after 24 h, far exceeding the 77.5–79.9% retention of TB‐HPBI‐140/180/220 (Figure 2c). Moreover, under extended testing (48 h) with varying Fe^2+^ concentrations (0.1–1 ppm), it maintained minimal degradation even as residual weight decreased from 97.7% to 88.4% with increasing radical concentration (Figure S8). This consistent stability across different test severities is consistent with the role of strengthened hydrogen bonding in mitigating free‑radical attack.

The strengthened hydrogen bonding contributed to the enhanced solvent resistance and PA stability of the membranes. While TB‐HPBI‐140 and TB‐HPBI‐220 remained soluble in hot DMSO (80°C) and exhibited unchanged ^1^H‐NMR spectra (Figure 1g), TB‐HPBI‐260 became completely insoluble in polar aprotic solvents such as *N*,*N*‑dimethylacetamide (DMAc) and *N*‑methyl‑2‑pyrrolidone (NMP) (Figure S9). A progressive solubility reduction in DMAc with increasing annealing temperature further corroborated the enhancement in hydrogen bonding (Figure S10). The membranes annealed at lower temperatures (140°C, 180°C, and 220°C) underwent rapid structural disintegration in 70 wt.% PA due to excessive swelling (Figure S11). In contrast, TB‐HPBI‐260 maintained structural integrity under the same conditions, forming a coherent gel phase with notable mechanical stability, demonstrated by a 250% elongation (increasing from 1.79 to 6.18 cm; Figure 2d). Systematic evaluation revealed substantially improved PA stability: at 70 wt.% PA doping, TB‐HPBI‐260 exhibited 445.8% uptake‐2.4 times that of TB‐HPBI‐140 under 65 wt.% PA doping. At 65 wt.% PA doping, it showed 154.2% uptake with only 15.1%‐dimensional swelling, significantly lower than that of the reference membrane under identical conditions (Table S2).

Strengthened hydrogen bonding significantly enhanced proton conductivity across 80°C–160°C (Figure 2e). Specifically, the TB‐HPBI‐260 membrane with 70 wt.% PA exhibited a conductivity of 125.7 mS cm^−^^1^ at 160°C under anhydrous conditions, which is 2.1 times higher than that of TB‐HPBI‐140 (58.6 mS cm^−^^1^). At 80°C, it reached 59.9 mS cm^−^^1^, marking a 6.7‐fold enhancement over TB‐HPBI‐140 (8.9 mS cm^−^^1^) and exceeding literature benchmarks [40]. Furthermore, even with 65 wt.% PA and reduced acid uptake, the TB‐HPBI‐260 membrane maintained superior proton conductivity compared to the TB‑HPBI‑140, ‑180, and ‑220 counterparts in the 80°C–160°C range.

Attributed to the enhanced proton conductivity, the membrane electrode assembly (MEA) fabricated with the TB‐HPBI‐260 membrane showed significantly improved performance, achieving a 27% higher peak power density (234.7 vs. 185.0 mW cm^−^^2^ at 160°C, H_2_/air) than its TB‐HPBI‐140 counterpart (Figure 2f). In addition, it exhibited remarkable operational durability. Under constant‐voltage operation (160°C, 0.3 V), performance stabilized after an initial decay phase (due to PA depletion [41]) and remained steady for more than 65 h (Figure 2g), despite the known degradation effects associated with standard operational cycles such as shutdowns and restarts [42, 43].

### 2.3 Fabrication of Amine–Anhydride Covalent Crosslinking Framework

The TBAm‐PBI was synthesized as illustrated in Figure S1b. Its structure was confirmed by ^1^H‐NMR (Figure S12), which revealed characteristic resonances: methylene protons of the TB unit at 4.23, 4.34, and 4.73 ppm; amine protons at 5.20 ppm; and two distinct benzimidazole ‐NH signals at 12.79 and 13.05 ppm, consistent with successful copolymerization. FT‐IR analysis (Figure S13) further confirmed the presence of N─H stretching vibration of ‐NH_2_ group at 3358 cm^−1^ and benzimidazole C═N/C═C stretching at 1631 cm^−1^. The novel TB‐derived dianhydride crosslinker (4*H*,9*H*,11*H*,13*H*‐8,17‐methanobenzo [4,5] isochromeno[6,7‐b] benzo [4,5] isochromeno[7,6‐f] [1,5] diazocine‐4,6,11,13(18*H*)‐tetraone, TB‐d) was synthesized via condensation of 4‐amino‐1,8‐naphthalic anhydride with dimethoxymethane (Figure S2), and its structure was verified by ^1^H NMR (Figure S14), FT‐IR (Figure S15), and elemental analysis (Table S3). Utilizing three dianhydrides (6FDA, NA, and TB‐d), covalently crosslinked networks were formed within TBAm‐PBI membranes via the amine‐anhydride reaction pathway (Figure 1a; Figure S1c). The resulting covalently crosslinked membranes were designated as TBAm‐PBI‐6F (crosslinked with 6FDA), TBAm‐PBI‐NA (crosslinked with NA), and TBAm‐PBI‐TB (crosslinked with TB‐d).

**FIGURE 3:**
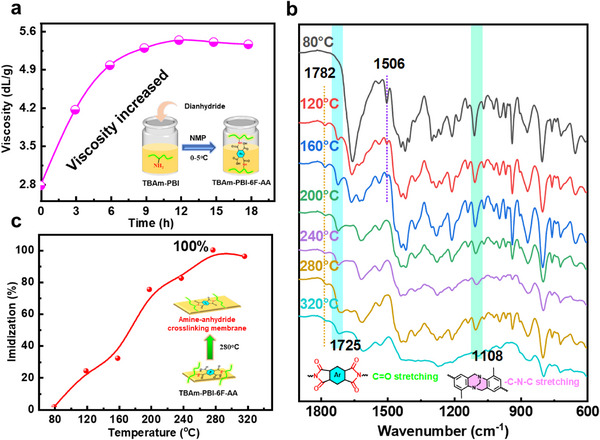
Crosslinking process of TBAm‐PBI‐6F membrane. a) Time‐dependent increase in the inherent viscosity of TBAm‐PBI after adding 6FDA, indicating rapid formation of the amic acid precursor TBAm‐PBI‐6F‐AA. b) FT‑IR spectra (600–1900 cm^−^^1^) of the TBAm‑PBI‑6F‐AA, showing the progression of imidization during thermal treatment. This process was monitored by FT‐IR, which showed the progressive disappearance of amic acid bands (e.g., amide I band C═O stretching at 1660 cm^−^^1^ and amide II band (N─H bending coupled with C─N stretching) at 1506 cm^−^^1^) above 200°C and the concurrent emergence of imide characteristic absorptions (e.g., C═O stretching at 1782 and 1725 cm^−^^1^, and C–N stretching at 1374 cm^−^^1^) above 120°C. c) The temperature‐dependent imidization degree of TBAm‑PBI‑6F‑AA was assessed via the FT‑IR intensity ratio of the imide C═O stretch (1725 cm^−^^1^) to the TB C─N─C vibration (1108 cm^−^^1^). The results demonstrate that imide ring formation is thermally driven and reaches completion at temperatures above 280°C.

Crosslinking was initiated by the formation of an amic acid precursor, namely TBAm‑PBI‑6F‑AA (from 6FDA), TBAm‑PBI‑NA‑AA (from NA), or TBAm‑PBI‑TB‑AA (from TB‑d) (Figure S1c). This precursor stage was followed by thermal imidization [44]. Using 6FDA as a representative crosslinker, the reaction with pendant amino groups was carried out in NMP at 0°C–5°C. The inherent viscosity of the solution increased rapidly from 2.73 to 4.14 dL g^−1^ within 3 h and plateaued after 12 h (Figure 3a). ^1^H NMR analysis of the membranes (Figure S12) demonstrated the successful reaction between amino groups and dianhydrides, as evidenced by the disappearance of the amine proton signal at 5.20 ppm and the broadening of the imidazole –N─H resonance into a band between 12.49 and 13.27 ppm. This conversion was further confirmed by FT‑IR spectroscopy (Figure S13), where the disappearance of the N─H stretching vibration of –NH_2_ at 3358 cm^−^^1^ and the emergence of a new amide II at 1506 cm^−^^1^ in the amic acid precursor were observed. An identical synthetic procedure was used to prepare TBAm‑PBI‑NA‑AA and TBAm‑PBI‑TB‑AA membranes with NA and TB‑d, respectively. The resulting amic acid precursor membranes were then thermally imidized under reduced pressure by heating from 80°C to 320°C.

The FT‐IR spectra in Figure 3b monitored the thermal imidization. As the temperature increased, the characteristic amic acid bands (amide I at 1660 cm^−^^1^ and amide II at 1506 cm^−^^1^) diminished progressively above 200°C. Concurrently, new bands characteristic of the imide structure emerged, notably the symmetric and asymmetric C═O stretches at 1782 and 1725 cm^−^^1^ and the C–N stretch at 1374 cm^−^^1^, which became evident above 120°C. The imidization degree, calculated from the intensity ratio of the imide asymmetric C═O stretching band (1725 cm^−^^1^) to the C─N stretching band of the TB units (1108 cm^−^^1^), reached completion at 280°C (Figure 3c). Corroborating this conversion, all resulting crosslinked membranes were insoluble in DMSO, NMP, DMAc, and 85% PA (Table S4), confirming the formation of a robust crosslinked network.

**FIGURE 4:**
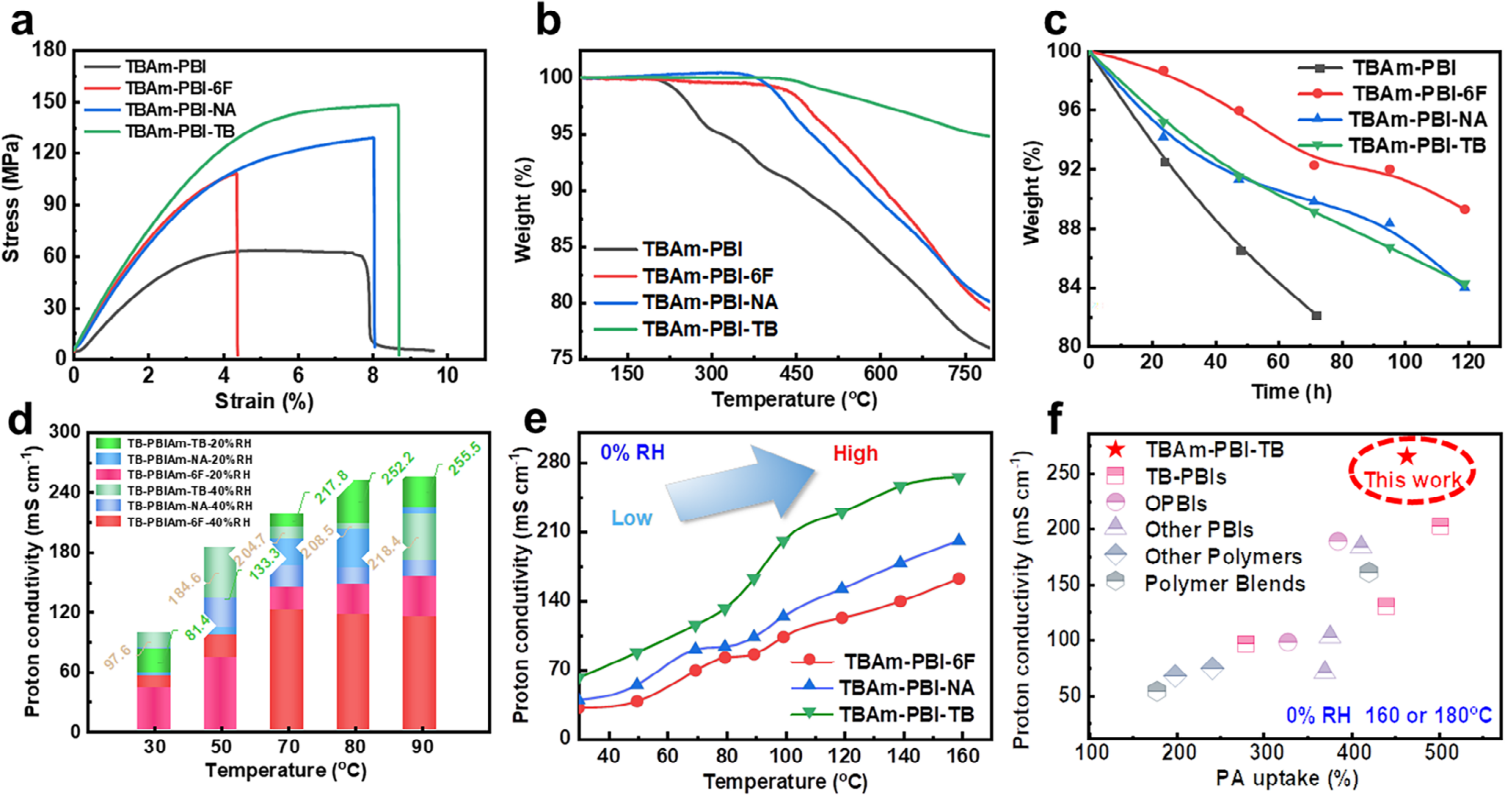
Properties of the TBAm‐PBI membrane and three amine–anhydride crosslinked membranes (TBAm‐PBI‐TB, TBAm‐PBI‐NA, and TBAm‐PBI‐6F). a) Mechanical properties of crosslinked membranes without PA doping. b) TGA thermograms of the TBAm‐PBI membrane and three amine–anhydride crosslinked membranes. c) Oxidative stability of the TBAm‐PBI membrane and three amine–anhydride crosslinked membranes. d) Temperature‐dependent proton conductivity of TBAm‐PBI‐TB, TBAm‐PBI‐NA, and TBAm‐PBI‐6F membranes under low RH. e) Anhydrous (0% RH) proton conductivity of PA‐doped TBAm‐PBI membranes across a wide temperature range (30°C–160°C), demonstrating effective proton conductivity without external humidification. f) Comparison of the high‐temperature proton conductivity (at 160°C or 180°C) of the TBAm‐PBI‐TB membrane with various high‐temperature PEMs (Table S5) reported in recent years.

As shown in Figure 4a, all covalently crosslinked membranes exhibited notably enhanced mechanical properties, with tensile strength and modulus reaching 107.5–147.7 MPa and 3.5–4.2 GPa, respectively, significantly exceeding those of non‑covalently crosslinked TBAm‑PBI (59.9 MPa, 2.59 GPa). With a tensile strength of 147.7 MPa, the TB‑d‑crosslinked membrane outperformed not only the 6FDA‐ and NA‐crosslinked membranes but also other reported counterparts (Table S5), owing to its denser hydrogen‑bonding network. TGA analysis revealed that covalent crosslinking substantially improved thermal stability, as reflected by the increase in the onset degradation temperature from 282 to 350°C (Figure 4b). In oxidative resistance tests (120 h Fenton's test; Figure 4c), the crosslinked membranes retained over 94.1% mass after 24 h and maintained structural integrity through 120 h, whereas non‐crosslinked TBAm‑PBI retained 92.5% and failed after 72 h. These enhancements collectively demonstrate the synergistic effect of hydrogen bonding and covalent crosslinking [21, 45, 46].

Acid uptake evaluation (80 wt.% PA, 12–24 h) showed that TBAm‐PBI underwent complete dissolution, while the corresponding crosslinked membranes retained their structural integrity despite swelling (Figure S16) [21, 46, 47]. Quantitative analysis (Table S6) indicated that covalently crosslinked TBAm‐PBI‐TB achieved the highest combined PA/water uptake (657.6%), outperforming TBAm‐PBI‐6F (430.9%) and TBAm‐PBI‐NA (594.3%), which can be ascribed to the increased basicity provided by the TB‐d. After dehydration at 80°C, TBAm‐PBI‐TB retained a PA uptake of 469.5%, supporting its ability to facilitate proton transport across a wide temperature range. Furthermore, the TBAm‐PBI‐TB membrane exhibited the highest swelling ratio (182.1%), with a dimensional increase from 1.03 to 1.73 cm (Figure S17). Consistent with these results, TBAm‐PBI‐TB also demonstrated a high PA retention of 67.8%, outperforming non‐crosslinked TBAm‐PBI (57.0%) and commercial *m*‐PBI (33.6%) (Table S7), underscoring its exceptional ability to retain acid.

### 2.4 Proton Conductivity and Fuel Cell Performance

The proton conductivity of the amine–dianhydride crosslinked membranes was measured from 30°C to 160°C under 0%–60% relative humidity (RH) (Figure 4d; Figure S18). Across 30°C–90°C and 20%–60% RH, these membranes showed conductivities of 42.4–255.5 mS cm^−^^1^ (Figure S18), highlighting their excellent low‐temperature/low‐humidity performance. The TBAm‐PBI‐TB membrane exhibited a high proton conductivity of 255.5 mS cm^−^^1^ at 90°C and 20% RH (Figure 4d). This performance significantly surpasses that of its counterparts, TBAm‐PBI‐6F (154.8 mS cm^−^^1^) and TBAm‐PBI‐NA (224.4 mS cm^−^^1^), and even exceeds Nafion 211 under fully hydrated conditions (145.2 mS cm^−^^1^ at 100% RH; Figure S19). Furthermore, under comparable conditions, its conductivity across 30°C–90°C and 20%–60% RH (81.4–255.5 mS cm^−^^1^) substantially exceeds the values reported for microporous copolyimides (e.g., 130 mS cm^−^^1^ for PI‐TB‐N40C at 90°C, 60% RH) and is over three orders of magnitude higher than that of sulfonated PBI (0.09 mS cm^−^^1^ at 65°C, 85% RH) [27, 31]. Such enhanced performance is attributed to an amine–anhydride crosslinked dual‐network structure that ensures high PA uptake, thereby facilitating efficient proton transport (Table S8). Arrhenius plots obtained at 20% RH (30°C–90°C) for the amine–anhydride crosslinked membranes gave proton conduction activation energies (*E*_a_) of 0.18–0.22 eV (Figure S20, Table S9), falling within the range characteristic of the Grotthuss mechanism [48]. Notably, proton conductivity did not improve monotonically with increasing RH (20%–60%). For example, the proton conductivity of TBAm‐PBI‐TB dropped from 255.5 to 218.4 mS cm^−^^1^ as RH increased from 20% to 40% (Figure S18). This non‐monotonic behavior may be explained by enhanced PA leaching under more humid conditions.

Under anhydrous conditions from 30°C to 160°C, the covalently amine–anhydride crosslinked membranes (TBAm‐PBI‐TB, TBAm‐PBI‐NA, and TBAm‐PBI‐6F) exhibited efficient proton conductivities of 29.0–264.7 mS cm^−^^1^ (Figure 4e). The low *E*_a_ values (0.07–0.19 eV, Figure S20 and Table S9) confirmed the Grotthuss‐type hopping mechanism. Owing to its high PA uptake capacity (469.5%), the TBAm‐PBI‐TB membrane showed the highest conductivity across the entire temperature range, reaching 264.7 mS cm^−^^1^ at 160°C. This performance substantially surpasses that of other reported TB‐based PA‐doped polymers and commercial Celtec‐P1000 (Figure 4f; Table S5) [28, 30, 31, 49, 50, 51, 52, 53, 54, 55, 56], highlighting the critical role of superior PA uptake in achieving exceptional anhydrous proton conductivity.

**FIGURE 5:**
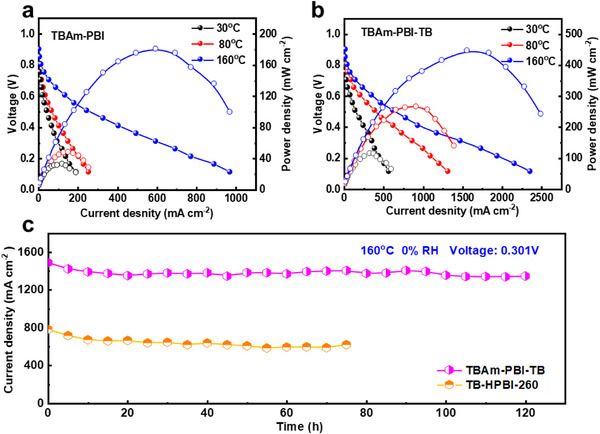
Fuel cell performance comparison between non‑covalently crosslinked TBAm‑PBI and amine‑dianhydride crosslinked TBAm‑PBI‑TB membranes. a,b) Polarization and power density curves measured at 30°C, 80°C, and 160°C under anhydrous H_2_/air conditions. c) Long‑term stability test at 160°C (anhydrous) for the TB‑HPBI‑260 and TBAm‑PBI‑TB membranes. The Pt loading on both anode and cathode (carbon paper electrodes) was 1.0 mg_p__t_ cm^−^^2^.

The MEAs based on covalently crosslinked PBI membranes delivered substantially higher peak power densities under anhydrous H_2_/air conditions across a wide temperature range of 30°C–160°C (Figure 5a,b; Figure S21). All fabricated MEAs consistently maintained open‐circuit voltages (OCVs) above 0.90 V, indicating effective gas barrier properties. As shown in Figure 5b, the MEA based on TBAm‐PBI‐TB achieved peak power densities of 108.6, 261.9, and 446.2 mW cm^−^^2^ at 30°C, 80°C, and 160°C, respectively. These values represent a significant enhancement over those of the *non*‐covalently crosslinked TBAm‐PBI (30.6, 45.8, and 180.3 mW cm^−^^2^ at the respective temperatures in Figure 5a) and surpass the performance of sulfonated PBI (122.2 mW cm^−^^2^ at 160°C) reported in the literature [27, 31]. The TBAm‐PBI‐TB membrane showed lower ohmic resistance at 160°C than the TBAm‐PBI‐6F and TBAm‐PBI‐NA membranes (Table S10); correspondingly, it achieved higher conductivity, current density, and power density. Furthermore, the power density at 160°C (446.2 mW cm^−^^2^) is substantially higher than the 367.8 mW cm^−^^2^ produced by a Nafion 211‐based MEA operated at 30°C and 40%–50% RH (Figure S22). This performance advantage is further underscored by its exceptional operational durability: a mere 9.54% loss in current density and a low decay rate of 1.18 mA cm^−^^2^ h^−^^1^ over 120 h at 160°C (Figure 5c). Consequently, its overall stability exceeds that of the TB‐HPBI‐260‐based MEA (Figure 2g).

## 3 Conclusion

In summary, we developed a novel Tröger's base‐functionalized PBI membrane featuring a dual‑network architecture that combines thermally reinforced hydrogen bonding with a robust amine–dianhydride covalent crosslinking framework. This synergistic design significantly improves PA uptake, proton conductivity, and mechano‑chemical stability. The optimized TBAm‑PBI‑TB dual‑network membrane exhibited proton conductivities of 255.5 mS cm^−^^1^ at 90°C (20% RH) and 264.7 mS cm^−^^1^ at 160°C under anhydrous conditions, and delivered excellent fuel‑cell performance over a wide temperature range of 30°C–160°C, surpassing conventional PBI and Nafion‑based membranes. The corresponding membrane electrode assembly delivered a peak power density of 446.2 mW cm^−^^2^ at 160°C, substantially higher than those of non‐covalently crosslinked and commercial benchmark membranes. Moreover, it demonstrated robust durability, exhibiting only a 9.54% current loss after 120 h of operation at 160°C. By employing a synergistic strategy based on hydrogen bonding and covalent crosslinking, this study introduces an approach to fabricate proton‐conducting membranes operable over a wide temperature range for use in anhydrous fuel cells. Future integration of techniques like 3D printing offers a promising route to advance these membranes, streamline their production, and transform the development of practical next‐generation fuel cells.

## 4 Experimental Section

### 4.1 Materials

Polyphosphoric acid (PPA), phosphorus pentoxide (P_2_O_5_), phosphoric acid (PA), dimethyl sulfoxide (DMSO), *N*,*N*‐dimethylformamide (DMF), trifluoroacetic acid (TFA, >99.0%), triethylamine, 5‐aminoisophthalic acid, 1,4,5,8‐naphthalenetetracarboxylic anhydride (NA), 4,4′‐(hexafluoroisopropylidene)diphthalic anhydride (6FDA), benzimidazole, and 4‐amino‐1,8‐naphthalic anhydride and diethoxymethane (DMM, 99.0%) were all purchased from Aladdin (China). 3,3′‐Diaminobenzidine (DAB, 99.0%) and 4‐amino‐3‐methylbenzoic acid were obtained from Tokyo Chemical Industry Co., Ltd. Methanol and ethanol used in this study were obtained from Energy Chemical Reagents Co., Ltd (China). All chemicals were used as received without further purification.

### 4.2 Synthesis of 4*H*,9*H*,11*H*,13*H*‐8,17‐Methanobenzo [4,5] Isochromeno[6,7‐b] Benzo [4,5] Isochromeno[7,6‐f] [1,5] Diazocine‐4,6,11,13(18*H*)‐Tetraone (TB‐d)

The synthesis of TB‐d was carried out through a one‐step reaction of 4‐amino‐1,8‐naphthalic anhydride with DMM in TFA, as outlined in Figure S2. Specifically, 4‐amino‐1,8‐naphthalic anhydride (2.13 g, 0.010 mol) was dissolved in DMM (5.2075 g, 0.050 mol) at room temperature under N_2_. After cooling the solution in an ice bath, TFA (30 mL) was added dropwise over 10 minutes. The mixture was subsequently stirred at room temperature for 48 h, leading to the gradual formation of a yellow precipitate. Finally, the solid product was isolated by filtration, washed repeatedly with acetic anhydride, and dried in a vacuum oven at 120°C for 12 h.

### 4.3 Synthesis of TB‐Based Polybenzimidazoles

The dicarboxylic acid monomer (TB‐COOH) bearing Tröger's Base unit was synthesized according to a previously reported procedure [31]. The Tröger's Base‐based polybenzimidazoles, TB‑HPBI and the novel amino‑functionalized derivative TBAm‑PBI, were synthesized via condensation polymerization in PPA following a reported procedure (Figure S1a,b) [57]. Typically, for the preparation of TBAm‑PBI, P_2_O_5_ (5 g) and PPA (40 g) were placed in a 100 mL three‑neck round‑bottom flask equipped with a mechanical stirrer under a nitrogen atmosphere. To this mixture, DAB (0.4285 g, 0.002 mol), TB‐COOH (0.6089 g, 0.0018 mol), and 5‐aminoisophthalic acid (0.0362 g, 0.0002 mol) were added. The reaction mixture was heated gradually to 110°C and maintained for 6 h, followed by sequential heating at 160°C for 12 h, 180°C for 12 h, and finally 200°C for an additional 12 h. The resulting viscous brown‐black polymer solution was cooled to room temperature and precipitated into a large volume of deionized water, yielding a fibrous solid. The polymer was collected by filtration, thoroughly washed with water and methanol until the filtrate was neutral, and dried under vacuum at 120°C for 12 h.

### 4.4 Formation of Amic Acid Precursors of Covalently Crosslinked Polybenzimidazoles

The synthesis of the amic acid precursors TBAm‑PBI‑6F‑AA, TBAm‑PBI‑NA‑AA, and TBAm‑PBI‑TB‑AA was carried out through a condensation reaction between the pendant amino groups of TBAm‐PBI and the dianhydride crosslinkers (6FDA, NA, TB‑d), maintaining the temperature at 0–5°C (Figure S1c). For example, the TBAm‐PBI‐6F‐AA was prepared as follows: TBAm‐PBI (0.0928 g) was dissolved in *N*‐methyl‐2‐pyrrolidone (NMP, 2.50 mL) at 80°C under nitrogen in a four‐neck flask. After complete dissolution, the solution was cooled to 0–5°C, and 6FDA (0.0044 g, 0.00001 mol) was added gradually under continuous stirring. The reaction was maintained at this temperature for 48 h, yielding the amic acid precursor. Following analogous synthetic protocols, the amic acid precursors of TBAm‑PBI‑TB and TBAm‑PBI‑NA were respectively prepared using TB‑d and NA under identical conditions.

### 4.5 Preparation of Amine–Dianhydride Covalent Crosslinking Polybenzimidazole Membranes

The amic acid precursor membranes were fabricated via solution casting. A polymer solution in NMP with a solid content of 4–5 wt.% was initially filtered through a 0.45 µm polytetrafluoroethylene (PTFE) syringe filter to remove insoluble particulates. The clarified solution was then cast onto a clean glass substrate and dried in a vacuum oven according to a stepwise temperature program: 30°C for 2 h, 60°C for 2 h, and 80°C for 1 h, yielding flexible yellow precursor membranes. To investigate the structural evolution during imidization, these precursor membranes were subjected to isothermal treatment in a muffle furnace under reduced pressure (‐0.1 MPa). The treatment was carried out at temperatures ranging from 80 to 320°C at 40°C intervals, with a holding time of 6 h at each temperature step.

### 4.6 Instruments and Characterizations

Nuclear magnetic resonance (NMR) spectra were recorded using an AVANCE Ш 600 MHz spectrometer (Bruker, Switzerland) with dimethyl sulfoxide‐*d*_6_ (DMSO‐*d*_6_) as the solvent. The thermal stability of polymer was evaluated by thermogravimetric analysis (TGA) using a TG‐DTA6300 instrument (NSK LTD, Tokyo, Japan). Samples were first heated to 150°C for 30 min to remove moisture. TGA curves were then recorded under nitrogen from 60 to 800°C at a heating rate of 10°C min^−1^. The mechanical properties of membrane samples (5 mm × 40 mm) with thickness of approximately 20 µm were measured using an AGS‐X‐10kN universal testing machine (Shimadzu, Japan) at a crosshead speed of 5 mm·min^−1^ at room temperature. Five specimens of each sample were tested. Polymer membranes were further characterized by Fourier transform infrared (FT‐IR) spectroscopy in attenuated total reflection‐transmission (ATR) mode using a Nicolet iS50 spectrometer (Thermo Fisher Scientific, USA). The inherent viscosity (*η*_inh_) of polymers was measured using a Ubbelohde viscometer. Elemental analysis was conducted with a Vario MACRO cube elemental analyser (Elementar Analysensysteme GmbH, Langenselbold, Hessen, Germany). Solid‐state ^1^H NMR (^1^H ssNMR) spectra were obtained using a Bruker 400MHz spectrometer, while ^1^H‐^1^ 2D NOESY spectrum of the polymers were recorded on an AVANCE Ш 600 MHz spectrometer in DMSO‐*d*6 solution. Dynamic mechanical analysis (DMA) was performed using a DMA Q800 (TA Instruments, USA) from 50 to 500°C at a heating rate of 10°C min^−1^. Differential scanning calorimetry (DSC) was carried out on a DSC Q2000 (TA Instruments, USA) to investigate the heat flow behaviour of the polymers.

### 4.7 Acid Doping and Dimensional/Weight Analysis

The dehydrated membranes were first characterized by their dry weight (*W*_dry_) and dry volume (*V*_dry_). Acid doping was carried out by immersing the membranes in PA solutions (65–80 wt.%) under ambient conditions for 72 h to control the acid doping level (ADL). After immersion, the membranes were removed, and any surface‐adherent acid was gently blotted off using filter paper. The weight and volume of the acid‐swollen membranes were then recorded as *W*_1_ and *V*_1_, respectively. Subsequently, the doped membranes were dried in an oven at 80°C for 2 h to determine the post‐doping dry weight, denoted as *W*_wet_.

The following parameters were calculated to quantify membrane behavior: the swelling ratio (SR), PA uptake, water uptake, and ADL. Here, *M*_PBI_ and *M*_PA_ represent the molecular weights of the PBI repeating unit and PA, respectively. The calculations were performed using Equations (1) to (4):

(1) $$\mathrm{SR}=\frac{\left(V_{1}-V_{\mathrm{dry}}\right)}{V_{\mathrm{dry}}}\times 100\%$$

(2) $$\mathrm{PA}\;\mathrm{uptake}=\frac{\left(W_{\mathrm{wet}}-W_{\mathrm{dry}}\right)}{W_{\mathrm{dry}}}\times 100\%$$

(3) $$\mathrm{Water}\;\mathrm{uptake}=\frac{\left(W_{1}-W_{\mathrm{wet}}\right)}{W_{\mathrm{dry}}}\times 100\%$$

(4) $$\mathrm{ADLs}=\frac{\left(W_{\mathrm{wet}}-W_{\mathrm{dry}}\right)}{W_{\mathrm{dry}}}\times \frac{M_{\mathrm{PBI}}}{M_{\mathrm{PA}}}$$

To evaluate the stability of the incorporated acid, the acid‐doped membranes were boiled in deionized water at 80°C for 24 h. The weight change before and after this treatment was used to calculate the PA retention.

### 4.8 Oxidative Stability Testing

The oxidative stability was evaluated using an accelerated test in Fenton's reagent (3 wt.% H_2_O_2_; Fe^2^^+^ concentrations: 0.1, 0.4, 0.7, 1.0, and 4.0 ppm) at 80°C. Pre‐weighed membrane samples (50–60 µm thick) were immersed in the reagent. Every 24 h, the membranes were removed, washed with deionized water, vacuum‐dried at 120°C for 6 h, and weighed. This process was repeated with fresh reagent for each cycle, and the cumulative weight loss was monitored over time.

### 4.9 Proton Conductivity Measurement

The proton conductivity of the PA‐doped membranes was determined by electrochemical impedance spectroscopy (EIS) using an electrochemical workstation (Metrohm Autolab PGSTAT302N). The measurements were performed over a frequency range of 0.1 Hz to 100 kHz. A rectangular membrane sample (1 cm × 3 cm) was mounted in a custom Teflon cell featuring a two‐electrode, in‐plane configuration with two platinum plates. The effective contact area was 1 cm^2^. The cell was placed in a temperature‐controlled chamber, and measurements were carried out from 30 to 160°C under various relative humidity (RH) conditions (0%, 20%, 40%, and 60% RH). The long‐term stability was assessed at 80°C and 20% RH. Prior to testing, all membranes were pre‐dried at 80°C for 2 h to remove residual water, ensuring the evaluation of conductivity under anhydrous conditions. The proton conductivity (*δ*) was calculated using Equation (5):

(5) $$\delta =\frac{L}{RWd}$$

where *δ* (in mS cm^−^^1^) denotes the proton conductivity, *L* (cm) is the distance between the two electrodes, *W* (cm) and *d* (cm) represent the width and thickness of the membrane, respectively, and *R* (Ω) is the measured resistance.

### 4.10 Single‐Cell Performance

Membrane electrode assemblies (MEAs) with an active area of 4.0 cm^2^ were fabricated by sandwiching the acid‐doped membrane between two gas diffusion electrodes (GDEs) without hot‐pressing. First, catalyst ink was prepared by dispersing 60 wt.% Pt/C (Johnson Matthey) in an aqueous isopropanol solution containing 5 wt.% PTFE. Subsequently, the ink was sprayed onto carbon paper substrates (HESEN Company) using an ultrasonic coating system (Sono‐Tek, W3586) to achieve a Pt loading of 1.0 mg_p__t_ cm^−^^2^ on both the anode and cathode. The single cell was operated with high‐purity H_2_ and compressed air at flow rates of 100 and 200 sccm, respectively. Polarization curves were recorded from 1.0 V to 0.1 V in 0.05 V steps using a KIKUSUI PLZ164WA test station across 80°C–160°C. Long‐term durability was assessed by monitoring the current density under a constant voltage of 0.3 V at 160°C.

### 4.11 Statistical Analysis

PA doping capability measurements were performed on three independently cast membranes for each formulation (*n* = 3), and the reported values represent the mean±standard deviation. Mechanical properties were measured on five independently cast membranes for each formulation (*n* = 5). All other characterization methods (e.g., TGA, FT‐IR, DMA, NMR, proton conductivity, fuel cell performance) were conducted as single measurements (*n* = 1), and representative data are shown. No formal hypothesis‐testing procedures were applied. Data processing and graphing were carried out using Origin, Chemdraw and Microsoft Excel.

## Funding

This study is supported by National Natural Science Foundation of China No. 52173210 (Y.B.Z.), Project Fund of Jiangsu Bingcheng Hydrogen Energy Technology Co., Ltd (Y.B.Z.), the financial support by the Nano·Materials Technology (Y.M.L.), Development Program (RS‐2023‐00235295) through the (NRF) funded by the Ministry of Science and ICT of South Korea (Y.M.L.), and the National Research Council of Science & Technology (NST) grant by the Ministry of Science and ICT of Korea No. GTL24052‐100 (Y.M.L.)

## Conflicts of Interest

J. Dai, J. Zhong, and Y. Zhuang have filed patent applications related to this manuscript (CN patent 202111006640X, 202511566097.7). All other authors declare no competing interests.

## Supporting information




**Supporting File**: advs74478‐sup‐0001‐SuppMat.docx.

## Data Availability

The data that support the findings of this study are available in the supplementary material of this article.
