# Supplementary material for: Synergistic Hydrogen‐Bonding and Covalent Crosslinking in Polybenzimidazole Membranes for Wide‐Temperature Anhydrous Fuel Cells
Source: Adv Sci (Weinh). 2026 Feb 24;13(24):e22161. doi: 10.1002/advs.202522161 (PMC13116360; doi:10.1002/advs.202522161)
Supplement: Supplementary file 1 — Supporting File: advs74478‐sup‐0001‐SuppMat.docx. [file ADVS-13-e22161-s001.docx]

Supporting Information

**Synergistic Hydrogen-Bonding and Covalent Crosslinking in Polybenzimidazole Membranes for Wide-Temperature Anhydrous Fuel Cells**

*Junming Dai†, Jianming Zhong†, Jinpeng Luo, Yuxing Song, Fan Hu, Young Moo Lee*, Yongbing Zhuang**

Junming Dai, Jianming Zhong, Jinpeng Luo, Yuxing Song, Fan Hu, Yongbing Zhuang

State Key Laboratory of Biopharmaceutical Preparation and Delivery, Institute of Process Engineering, Chinese Academy of Sciences, Beijing 100190, China
E-mail: ybzhuang@ipe.ac.cn (Y. Zhuang)

Junming Dai, Jianming Zhong, Jinpeng Luo, Fan Hu, Yongbing Zhuang
School of Chemical Engineering, University of Chinese Academy of Sciences, Beijing 100049, China

Young Moo Lee
Department of Energy Engineering, College of Engineering, Hanyang University, Seoul 04763, Republic of Korea

E-mail: ymlee@hanyang.ac.kr (Y.M. Lee)

† Junming Dai and Jianming Zhong contributed equally to this work.

**Molecular Simulation**

**Strengthened N–H⋯N hydrogen bonding interaction.** The molecular simulations were carried out using the GROMACS package (version 2021.3)^[1–4]^. Molecular geometries were initially optimized in Gaussian 16. The simulation systems were constructed using Packmol^[5]^, comprising 50 molecules in a cubic box with the edge size of 5nm. Atomic interactions were described using the OPALS-AA (Optimized Potentials for Liquid Simulations-All-Atom) force field^[6]^, and RESP charge obtained from Multiwfn^[7]^. Following energy minimization, the systems were equilibrated in the NPT ensemble using the Berendsen method for 2ns. Production MD simulations were then performed in the NPT ensemble at designated temperatures with a time step of 1 fs. The system temperature was controlled at 140°C and 260°C using a V-rescale thermostat (τ_T_=1ps). After 20ns of simulation, the number of hydrogen bonds were analysed using built-in GROMACS analysis tools.

**The proton affinity and proton transfer energy barrier of TB group.** The electrostatic potential distribution, and molecule geometries of all geometrical optimization were calculated using Becke’s three-parameter exchange functional^[8]^ combined with the Lee-Yang-Parr correlation functional^[9]^ (B3LYP) with Grimme's DFT-D3(BJ)^[10]^ empirical dispersion correction, and the 6-31 + +G(d,p) basis set. The neutral molecule (B) was calculated as the negative of the enthalpy change based upon a mole of particles for the following reaction^[11]^.

B + H^+^ = BH^+^

The geometries of all molecules been optimized and no imaginary frequencies for the harmonic frequency. Transition state (TS) structures were located using the TS optimization algorithm with Berny optimization in redundant internal coordinates. The TS structures were confirmed by the presence of one and only one imaginary frequency corresponding to the reaction coordinate. To verify the connection between reactants, transition states, and products, intrinsic reaction coordinate (IRC)^[12]^ calculations were performed in both forward and reverse directions from each transition state using the local quadratic approximation (LQA) method with a maximum of 20 points in each direction.The DFT calculations were performed on Gaussian16 program.


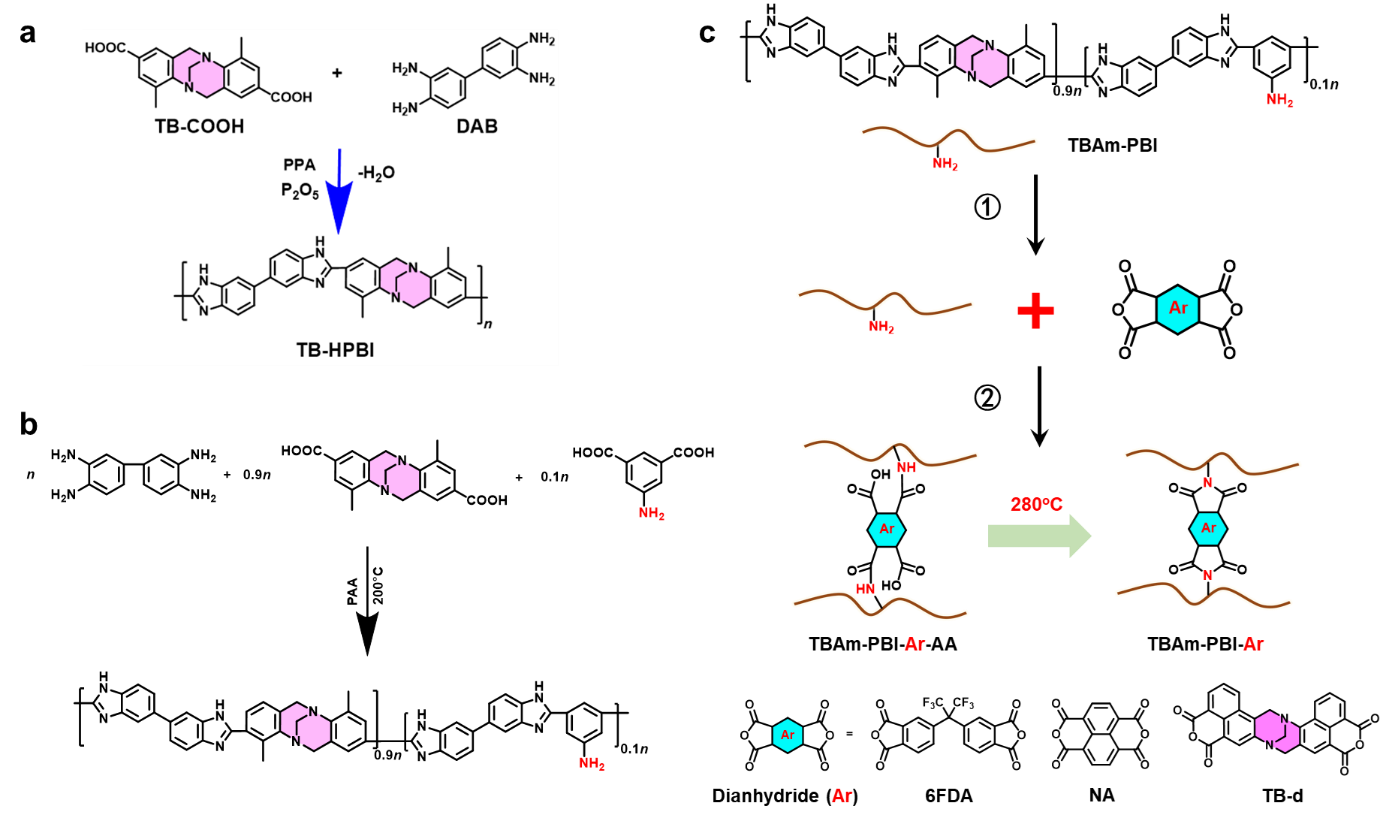


**Figure S1.** (a) Synthesis route of TB‑HPBI. (b) Synthesis route of TBAm‑PBI. (c) Fabrication of the amine‑anhydride covalent crosslinking framework. The crosslinked networks were fabricated by reacting TBAm‑PBI with three dianhydrides (6FDA, NA, and TB‑d). First, the reaction produced an amic acid precursor, namely TBAm‑PBI‑6F‑AA, TBAm‑PBI‑NA‑AA, or TBAm‑PBI‑TB‑AA. Subsequently, these intermediates yielded the final covalently crosslinked membranes, which were designated as TBAm‑PBI‑6F, TBAm‑PBI‑NA, and TBAm‑PBI‑TB, corresponding to the dianhydride used.


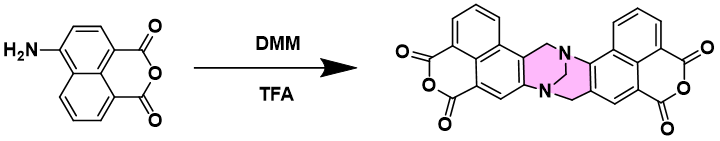


**Figure S2.** Synthesis of novel 4*H*,9*H*,11*H*,13*H*-8,17-methanobenzo [4,5] isochromeno[6,7-b] benzo [4,5] isochromeno[7,6-f] [1,5] diazocine-4,6,11,13(18*H*)-tetraone (TB-d).


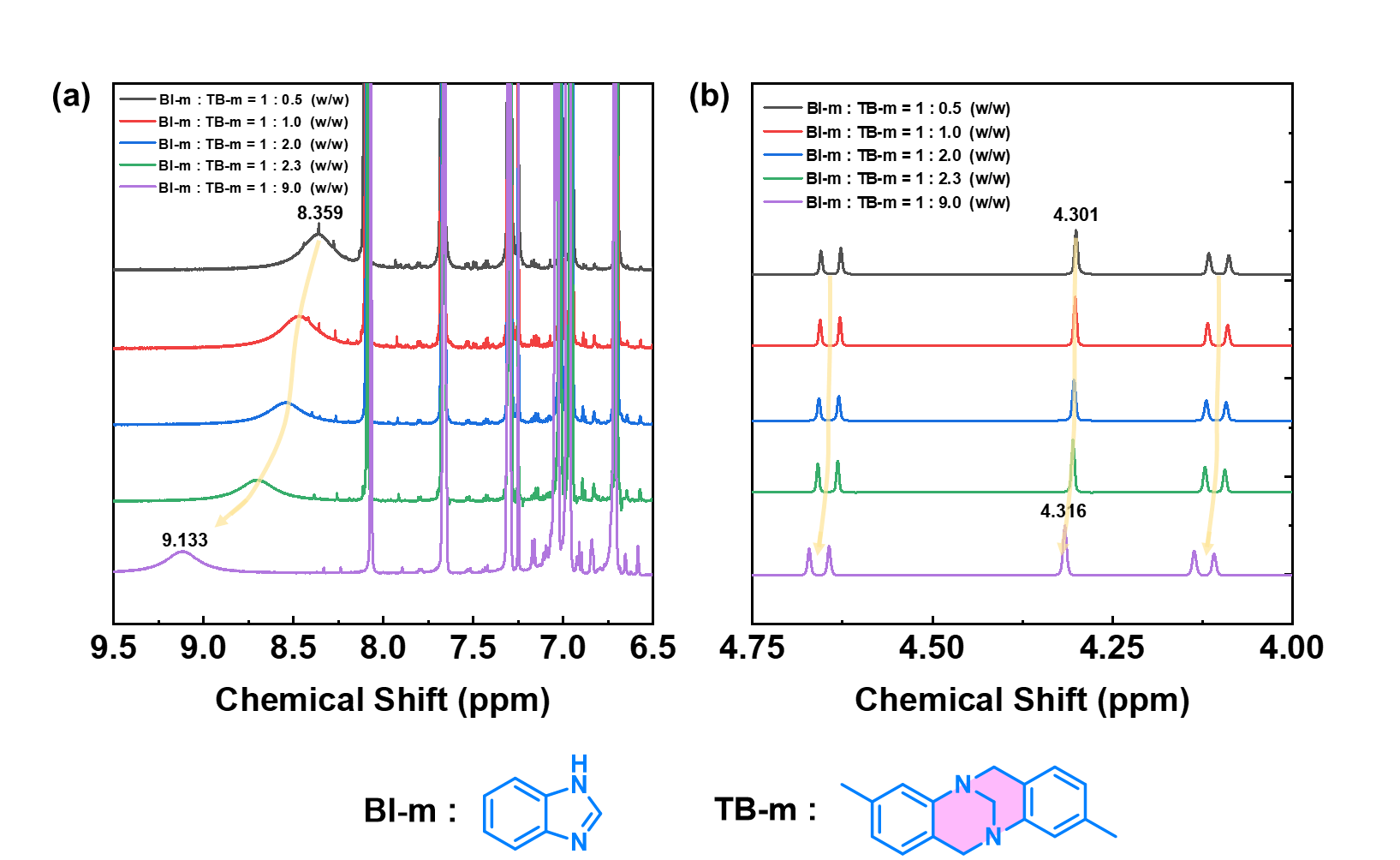


**Figure S3.** ¹H NMR spectra of BI-m and TB-m blends at varying ratios, recorded in chloroform‑*d*_6_. The corresponding spectral regions of 9.50–6.50 ppm and 4.75–4.00 ppm are displayed in panels (a) and (b), respectively. Increasing the content of TB-m resulted in a marked downfield shift of the N–H proton signal in BI-m, from 8.359 to 9.133 ppm. Concurrent downfield shifts were observed for the protons of the TB moiety, as exemplified by the bridgehead methylene protons moving from 4.301 to 4.316 ppm. These coordinated changes suggest an altered electronic environment, which is attributed to the enhanced hydrogen-bonding interactions between TB-m and BI-m.





**Figure S4.**  Solid-state ¹H NMR spectra were acquired for pure BI‑m, pure TB‑m, and their 7:3 (w/w) blend. In pure TB‑m, the N–H proton of the benzimidazole unit exhibited a broad, weak signal at 13.9 ppm, consistent with strong intermolecular hydrogen bonding. The bridgehead methylene protons appeared as a singlet, while the *exo* and *endo* protons of the remaining methylene units merged into a single resonance. Upon blending TB‑m with BI‑m, distinct spectral changes were observed: the benzimidazole N–H proton signal split into two resolved resonances at 13.0 and 14.2 ppm, indicating partial disruption of the original intermolecular hydrogen-bonding network due to newly formed interactions between the benzimidazole units of TB‑m and the TB groups. Concurrently, the two methylene poton signals (2.94 and 4.54 ppm) associated with the TB group coalesced into a single broad envelope centered near 3.6 ppm, providing additional evidence for strong inter‑component interactions within the blend^[13,14]^.


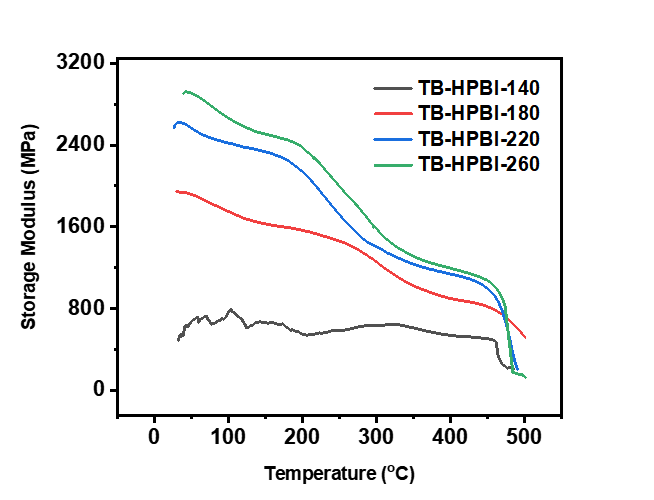


**Figure S5.**  Storage modulus of annealed TB-HPBI membranes from DMA.


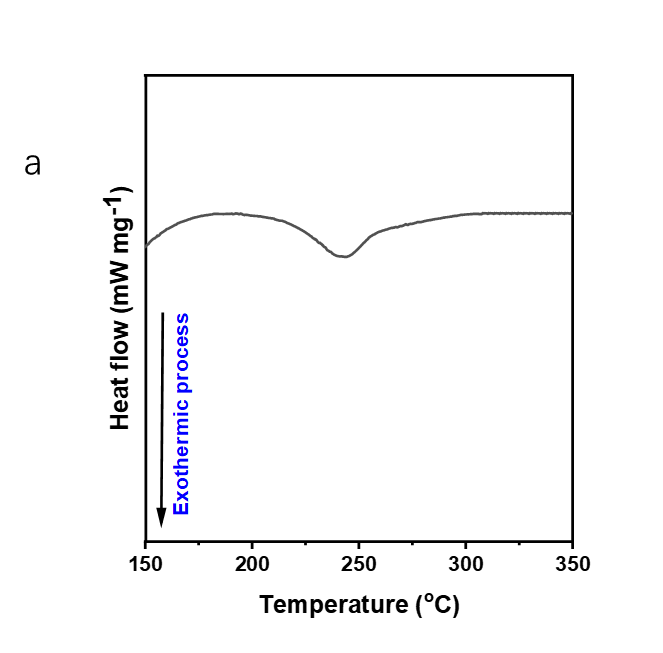


**Figure S6.** DSC curve of the TB-HPBI membrane reveals a distinct exothermic event, which is consistent with the formation of hydrogen bonds.


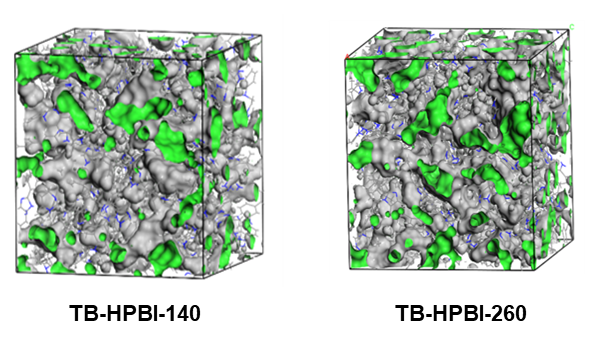


**Figure S7.** Strengthened N–H⋯N hydrogen bonding interactions. Three-dimensional structural models of TB-HPBI-140 and TB-HPBI-260, illustrating differences in molecular packing and hydrogen-bond networks.


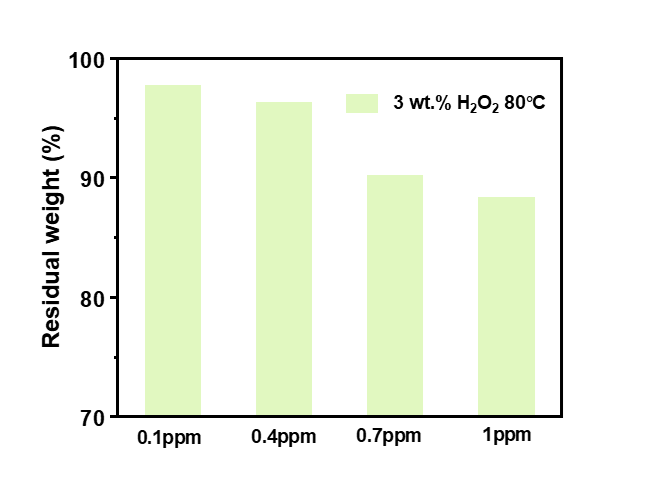


**Figure S8.** Residual weight of TB-HPBI-260 varied across the tested Fe²⁺ concentration range of 0.1 to 1 ppm.


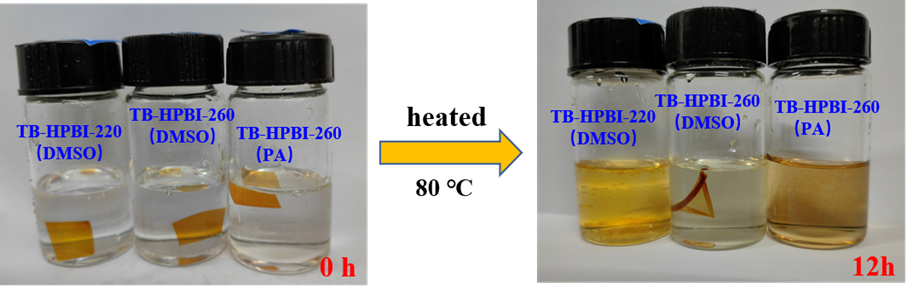


**Figure S9.** Solubility of TB‑HPBI‑220 and TB‑HPBI‑260 in DMSO and 85 wt.% PA solution. The TB‑HPBI‑220 membrane dissolved in DMSO after heating at 80°C for 12 h, whereas the TB‑HPBI‑260 membrane remained insoluble in DMSO under the same conditions but dissolved in PA solution. This indicates that TB‑HPBI‑260 possesses strong hydrogen‑bonding interactions that are disrupted in PA, and that it remains non‑crosslinked even after 260°C annealing.





**Figure S10.** Solubility comparison of annealed membranes in DMAc.


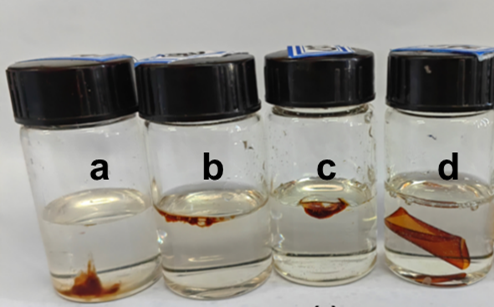


**Figure S11.** Morphological images show the annealed membranes doped with 70 wt.% phosphoric acid solution: (a) TB-HPBI-140, (b) TB-HPBI-180, (c) TB-HPBI-220, and d) TB-HPBI-260. It was observed that the TB-HPBI-140, TB-HPBI-180, and TB-HPBI-220 membranes exhibited loss of integrity from excessive swelling, whereas the TB-HPBI-260 membrane remained intact.





**Figure S12.** ¹H NMR spectra of TBAm–PBI versus its 6FDA-derived amic acid precursor TBAm-PBI-6F-AA in DMSO-*d*₆. The -NH₂ proton signal at 5.20 ppm in TBAm-PBI disappeared after reaction with 6FDA. Simultaneously, the doublet at 12.79/13.05 ppm (amic acid -NH-) transformed into a broad singlet spanning 12.49–13.27 ppm, confirming the reaction between amino groups and dianhydride.





**Figure S13.** Comparison of the FT-IR spectra of TBAm–PBI and its amic acid precursor, TBAm-PBI-6F-AA, reveals key changes indicative of the reaction. The characteristic N-H stretching band at 3358 cm⁻¹ observed in TBAm-PBI disappeared following its reaction with 6FDA. Correspondingly, a new absorption band appeared at 1506 cm⁻¹ in the spectrum of TBAm-PBI-6F-AA, which is attributed to the amide II vibration (resulting from the coupling of N-H bending and C-N stretching). This spectral evolution confirms the successful formation of the amic acid structure (TBAm-PBI-6F-AA).


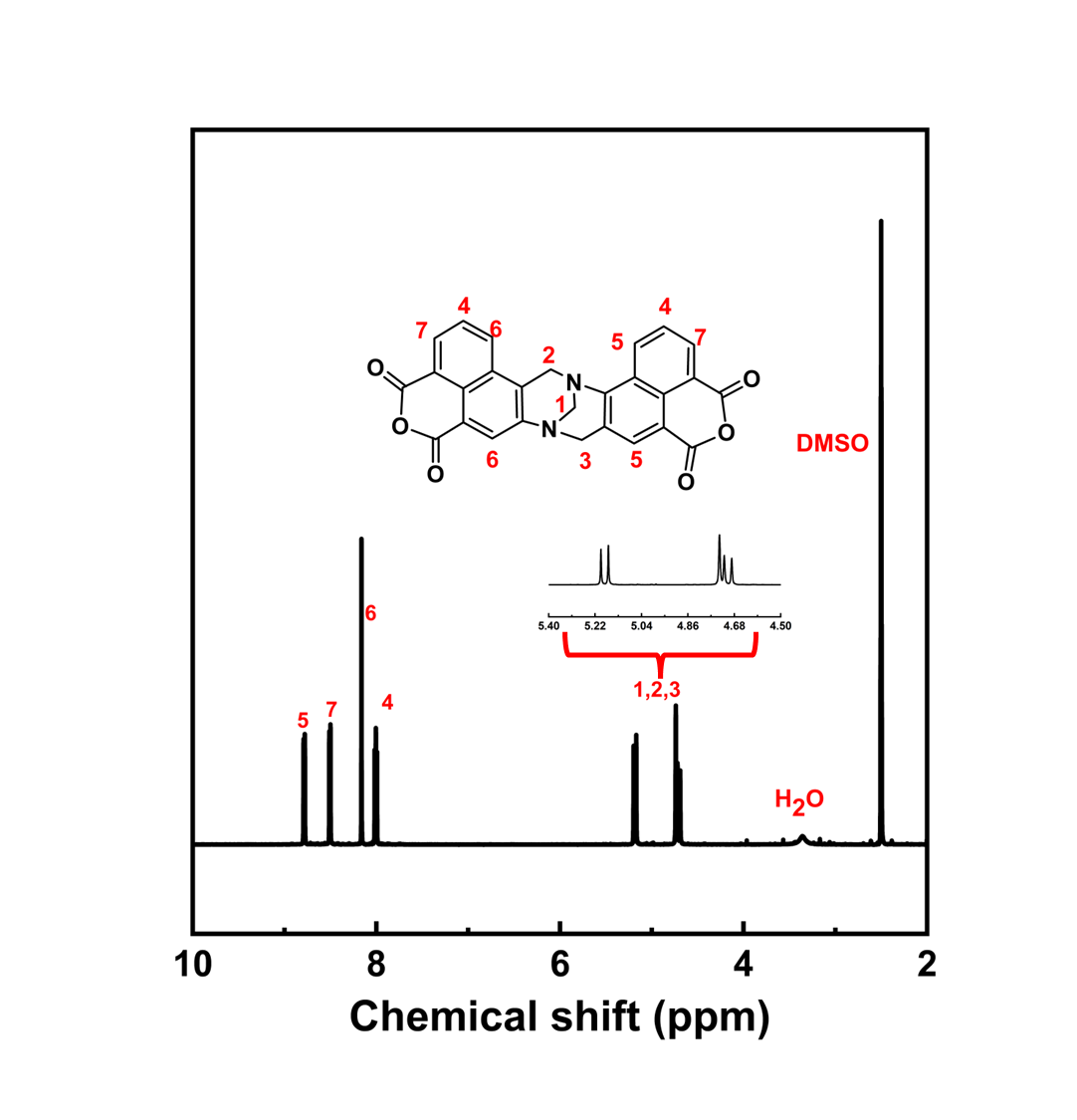


**Figure S14.** ^1^H NMR spectrum of TB-d monomer (in DMSO‑*d*₆).





**Figure S15.** FT-IR spectrum of the synthesized TB-d monomer.


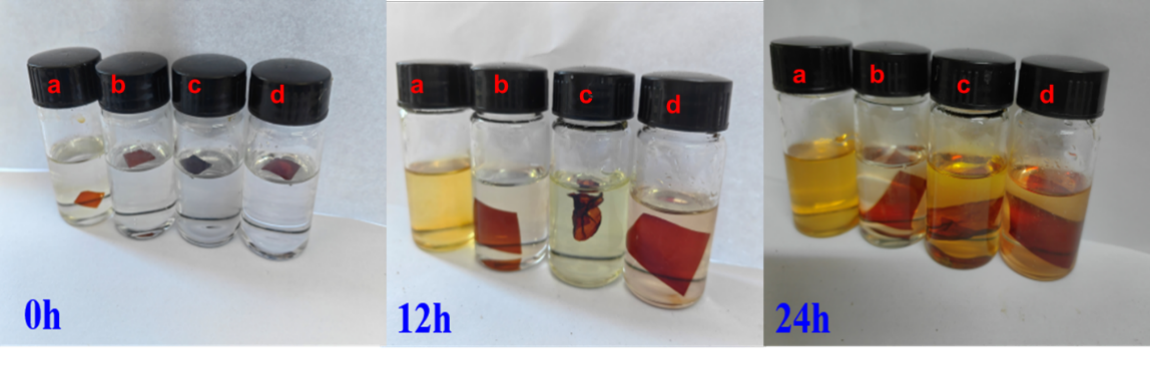


**Figure S16.** Stability evaluation of the membranes in 80 wt.% phosphoric acid at 0, 12, and 24 h: (a) TBAm-PBI, (b) TBAm-PBI-6F, (c) TBAm-PBI-NA, d) TBAm-PBI-TB. While the TBAm-PBI membrane dissolved at 12 h, the crosslinked membranes (b-d) remained stable with only swelling observed throughout the 24 h test.


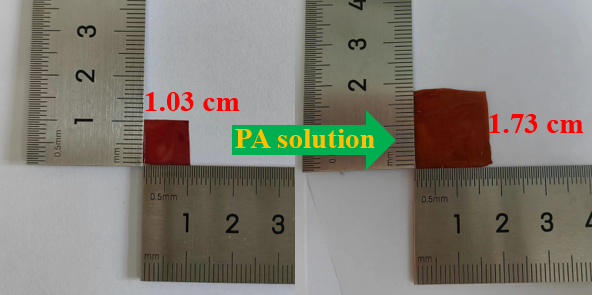


**Figure S17.** Morphological changes of the TBAm-PBI-TB membrane before and after immersion in phosphoric acid (PA) were examined. Upon exposure to an 80 wt.% PA solution, the membrane underwent significant swelling, exhibiting a linear expansion of approximately 70% and an area expansion of approximately 300%.

**
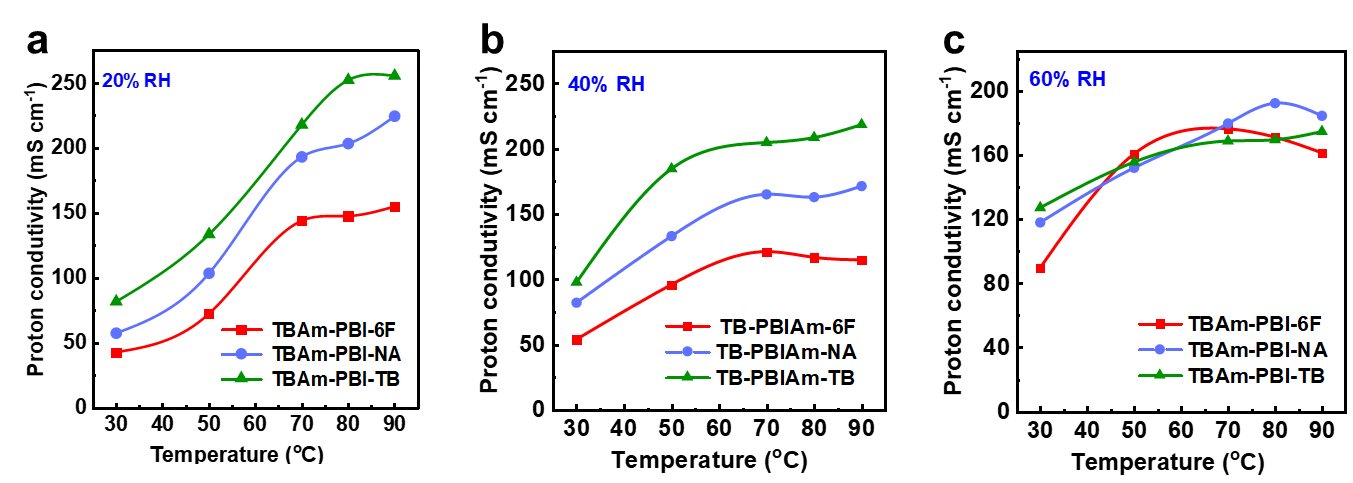
**

**Figure S18.** Proton conductivity of the amine–anhydride crosslinked membranes doped with PA. Panels (a–c) show the temperature-dependent proton conductivity of TBAm-PBI-6F, TBAm-PBI-NA, and TBAm-PBI-TB membranes measured from 30 to 90°C under low relative humidity conditions: (a) 20 % RH, (b) 40 % RH, and (c) 60 % RH.

**Figure S19.** Proton conductivity of the Nafion® 211 membrane (25 μm) under fully humidified conditions (100% RH).


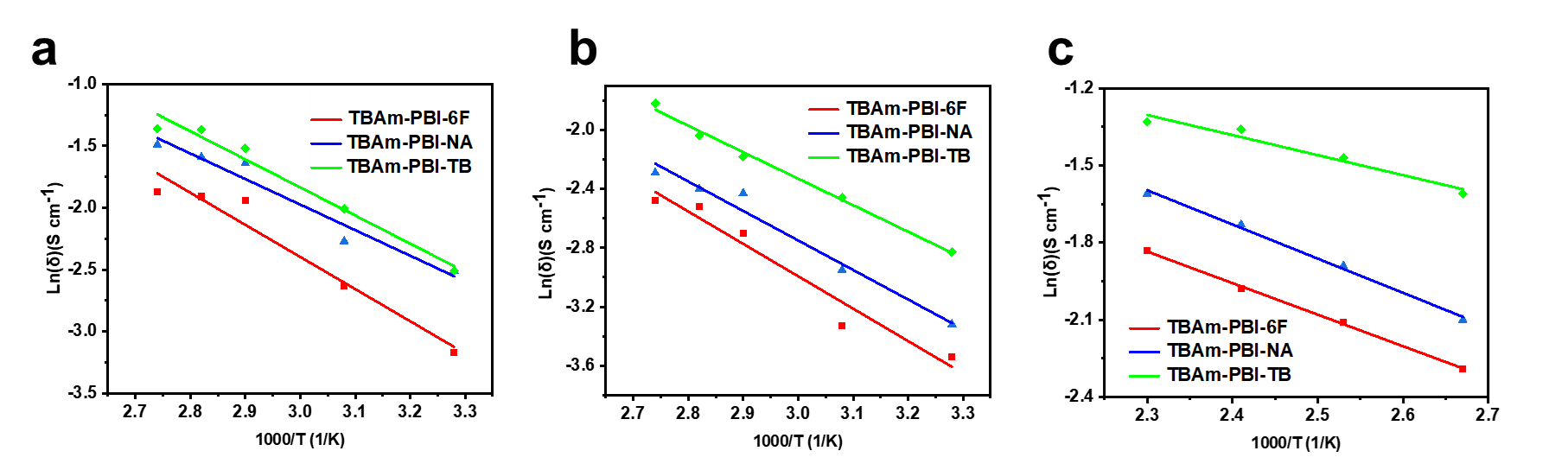


**Figure S20**. Arrhenius plots of the PA-doped crosslinked membranes: (a) from 30 to 90°C under 20% RH; (b) from 30 to 90°C under anhydrous conditions; (c) from 100 to 160°C under anhydrous conditions.


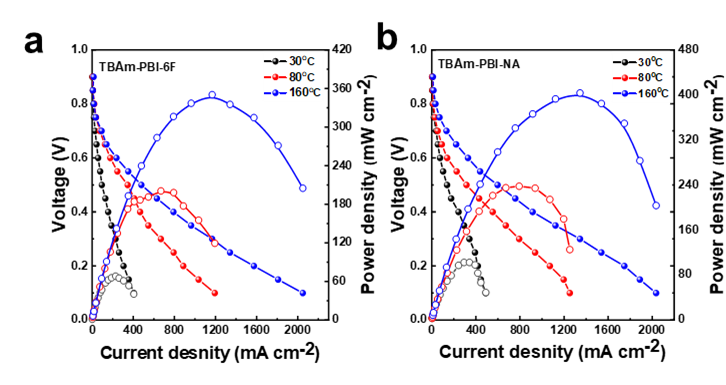


**Figure S21.** Fuel cell performance of amine–dianhydride crosslinked membranes. Polarization and power density curves under H₂/air at 30°C, 80°C, and 160°C under anhydrous conditions for cells assembled with (a) TBAm-PBI-6F and (b) TBAm-PBI-NA membranes.


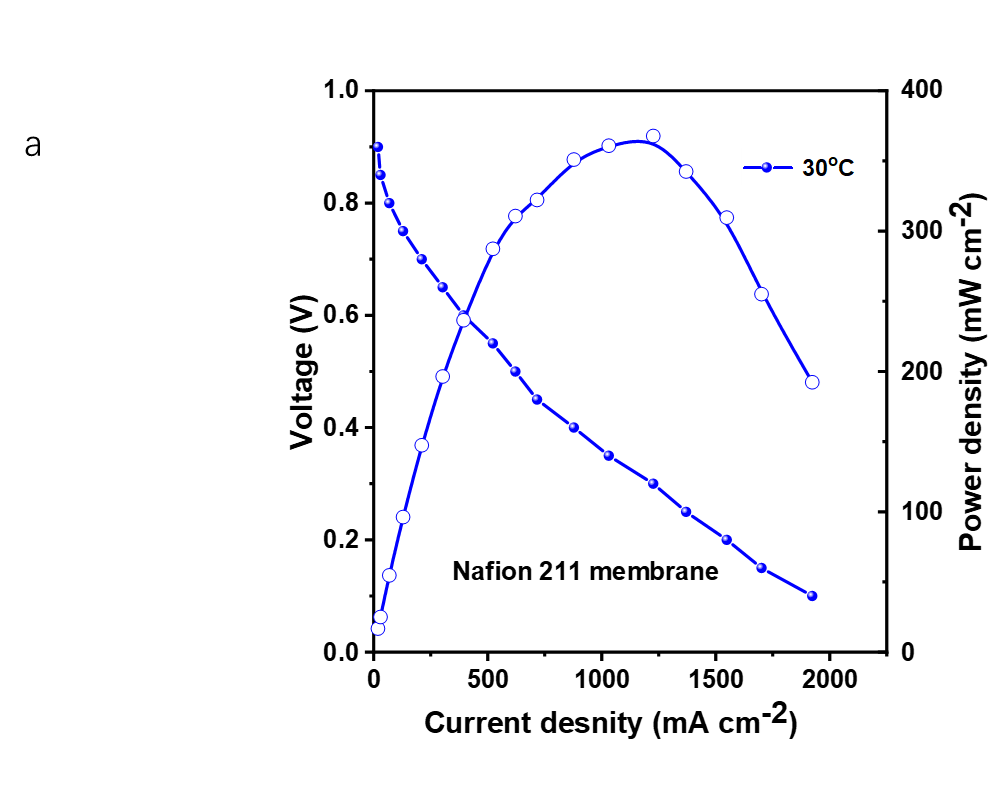


**Figure S22.** Polarization curve and power density of a Nafion 211 membrane were evaluated in an H₂/air fuel cell operated at 30°C under ambient relative humidity (40–50% RH).

**Table S1.** Elemental analysis of the TB-HPBI-180 and TB-HPBI-260 membranes

| **Category** | **C** | **H** | **N** |
| --- | --- | --- | --- |
| TB-HPBI-180 | 70.05 | 5.74 | 14.89 |
| TB-HPBI-260 | 71.71 | 5.48 | 15.37 |

**Table S2.** Key parameters, namely phosphoric acid (PA) uptake, acid doping level (ADL), and area swelling, were determined to characterize the PA absorption by the polymer membranes after immersion in PA solution

| **Category** | **PA**  **Solution** | **PA uptake (%)** | **Area Swelling (%)** | **ADL** |
| --- | --- | --- | --- | --- |
| TB-HPBI-140 | 65 wt.% | 188.0±7.2% | 18.8±0.6% | 9.19±0.34 |
| TB-HPBI-180 | 65 wt.% | 178.2±12.4% | 18.0±0.4% | 8.71±0.60 |
| TB-HPBI-220 | 65 wt.% | 170.5±5.2% | 17.1±0.2% | 8.33±0.56 |
| TB-HPBI-260 | 65 wt.% | 154.2±9.2% | 15.1±0.2% | 7.53±0.80 |
| TB-HPBI-260 | 70 wt.% | 445.8±23% | 84.2±0.7% | 21.79±4.50 |

**Table S3.** Elemental analysis of synthesized TB-d monomer

| **Category** | **C** | **H** | **N** |
| --- | --- | --- | --- |
| Analytical Calculated | 70.13 | 3.05 | 6.06 |
| Found | 69.8 | 2.69 | 5.96 |

**Table S4.** Solubility of TBAm-PBI and amine-anhydride crosslinked membranes

| **PBI^[a]^** | **Solvent** | | | | | |
| --- | --- | --- | --- | --- | --- | --- |
|  | **DMF^[b]^** | **DMAC^[c]^** | **NMP[^d]^** | **DMSO^[e]^** | **PA^[f]^** | **MSA^[g]^** |
| TBAm-PBI | **+** | **++** | **++** | **++** | **++** | **++** |
| TBAm-PBI-6F | **-** | **-** | **-** | **-** | **-** | **-** |
| TBAm-PBI-NA | **-** | **-** | **-** | **-** | **-** | **-** |
| TBAm-PBI-TB | **-** | **-** | **-** | **-** | **-** | **-** |

^[a]^The solubility was determined by using 3% solid content sample in solvent. The symbols represent the following: ++, soluble at room temperature; +, partial soluble at 80^o^C; -, insoluble at 80^o^C.

^[b]^DMF: *N*,*N*-dimethylformamide;

^[c]^DMAC: *N*, *N*-dimethyl acetamide;

^[d]^NMP: *N*-methyl-2-pyrrolidone;

^[e]^DMSO: dimethyl sulfoxide;

^[f]^PA: 85wt.% phosphoric acid;

^[g]^MSA: methanesulfonic acid.

**Table S5.** Comparison of the high-temperature proton conductivity (at 160 or 180°C) of the TBAm-PBI-TB membrane with various PEMs reported in recent years

| **Category** | **Polymers** | **Tensile stress^[^*^a^*^]^ (MPa)** | **Elongation^[^*^a^*^]^ (%)** | **PA uptake (%)** | **Proton conductivity**  **(mS cm^-1^)** | **Reference** |
| --- | --- | --- | --- | --- | --- | --- |
| OPBIs | CPyOPBI-OH-20 | 102.7 | 16.8 | 331 | 95 | [15] |
|  | CLx-OPBI | - | - | 389 | 187 | [16] |
| Other OPBIs | AGPBI | 71.5 | 11.1 | 374 | 67 | [17] |
|  | Nitrogen-Rich PBI | 72.62 | 7.46 | 380.3 | 99.6 | [18] |
|  | Cation-rich domain PBI | 115.2 | 54.8 | 416 | 181.6 | [19] |
| Other Polymers | CL-5% pECH | - | - | 201 | 64 | [20] |
|  | PI@PPy | 135.1 | 7.9 | 244 | 71 | [21] |
| Polymer Blends | OPBI/copolymer | - | - | 180 | 50 | [22] |
|  | DMBP-TB/PEKC | - | - | 425 | 159 | [23] |
| TB-PBIs | TB-PBI-50 | 146.9 | 26.1 | 283 | 93.2 | [24] |
|  | TB-N-HPBI | 96 | 3.8 | 508 | 201 | [25] |
|  | TB-HPBI-260 | 157.2 | 7.6 | 445.8 | 127.5 | This work |
|  | TBAm-PBI-TB | 147.7 | 8.8 | 469.5 | 264.7 | This work |

**^[^*^a^*^]^** Without PA doping

**Table S6.** PA and H_2_O uptake capability of amine-anhydride crosslinked membranes

| **Category** | **PA+H_2_O uptake^[a]^ (%)** | **PA uptake^[b]^ (%)** | **Water uptake^[c]^ (%)** | **Swelling ratio^[d]^ (%)** | **Thickness change (μm)** |
| --- | --- | --- | --- | --- | --- |
| TBAm-PBI-6F | 430.9±7.7% | 318.3 ±5.9% | 112.6±1.9% | 134.0±2.4% | 49/96 |
| TBAm-PBI-NA | 594.3±4.1% | 405.7±4.1% | 186.8±4.7% | 147.8±1.5% | 65/125 |
| TBAm-PBI-TB | 657.6±5.7% | 469.5±7.6% | 188.1±4.7% | 182.1±2.7% | 53/103 |

^[a]^Uptake was measured with 80 wt.% PA solution at 25°C.

^[b]^ The PA uptake was measured after drying the membranes at 80°C for 2 h to remove absorbed water.

^[c]^ Water uptake was calculated via the weight change of the membranes after drying at 80°C for 2 h.

^[d]^Swelling ratio was measured by the change of membrane volume after 80wt.% PA doping at 25^o^C.

**Table S7.** PA retention of the prepared membranes

| **Category** | **PA retention (%)** |
| --- | --- |
| mPBI | 33.6 |
| TBAm-PBI | 57.0 |
| TBAm-PBI-TB | 67.8 |

**Table S8.** Comparison of proton affinity values and energy barriers for various proton transfer pathways

| Molecule or PT pathways | Proton affinity  (kJ·mol^-1^)^[a]^ | Energy barrier  (kJ·mol^-1^)^[b]^ | **Reference** |
| --- | --- | --- | --- |
| Benzimidazole | 951.7 | - | [26] |
| H_2_PO_4_^-^ | 1359.7 | - | [26] |
| H_3_PO_4_ | 822.6 | - | [26] |
| H_2_O | 683.9 | - | [26] |
| TB^[c]^ | 958.6 |  | This work |
| Benzimidazole cation → benzimidazole | - | 4.1 | [26] |
| Benzi midazole → benzimidazole | - | 113.9 | [26] |
| Benzimidazole cation → H_3_PO_4_ | - | 69.6 | [26] |
| H_3_PO_4_ → benzimidazole | - | 114.4 | [26] |
| Benzimidazole → H_2_PO_4_^−^ | - | 36.5 | [26] |
| TB cation →TB^[c]^ | - | 6.2 | This work |
| TB cation → benzimidazole^[c]^ | - | 9.7 | This work |
| TB cation → H_3_PO_4_ | - | / | This work |
| H_4_PO_4_^+^ → H_3_PO_4_ | - | 4.9 | [26] |
| H_3_PO_4_ → H_2_PO_4_^−^ | - | 16.0 | [26] |
| H_3_O^+^ → H_3_PO_4_ | - | 0 | [27] |

^[a]^ A higher proton affinity indicates a stronger tendency to bind protons. In a PA-doped PBI membrane, dihydrogen phosphate anion (H₂PO₄⁻) is considered as the proton carrier. The proton affinity of H₂PO₄⁻ is 1359.7 kJ·mol⁻¹, substantially higher than that of TB (958.6 kJ·mol⁻¹) and benzimidazole (951.7 kJ·mol⁻¹), signifying its stronger proton-binding capability. Accordingly, if PA doping levels are high enough, protons dissociated from PA are more readily captured by H₂PO₄⁻ than by the TB units and benzimidazole groups on PBIs^[26]^.

^[b]^ Based on the energy barriers summarized above, the most favorable proton transfer (PT) pathway under high PA doping involves free PA molecules and their corresponding ions^[26]^. Thus, in anhydrous or low-humidity membranes with high PA content, proton conduction is primarily governed by a Grotthuss-type hopping mechanism within the crosslinked PBI network^[28]^.

^[c]^ The chemical structures of the theoretical models established for the simulation calculations:

.
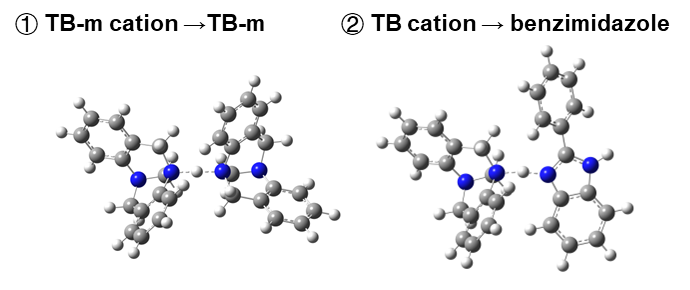


TB cation → benzimidazole TB cation →TB

**Table S9.** Proton conductivity activation energy (*E*_a_) of amine-anhydride crosslinked membranes

| **Polymers** | ***E*_a1_^[a]^ (eV)** | ***E*_a2_^[b]^** | ***E*_a3_^[c]^** |
| --- | --- | --- | --- |
| TBAm-PBI-6F^d^ | 0.22 | 0.19 | 0.11 |
| TBAm-PBI-NA^d^ | 0.18 | 0.17 | 0.11 |
| TBAm-PBI-TB^d^ | 0.18 | 0.15 | 0.07 |

^[a]^ The data is derived from proton conductivity under conditions of 30-90°C and 20% RH.

^[b]^ The data is derived from proton conductivity at 30-90°C and 0% RH.

^[c]^ The data is derived from proton conductivity at 100-160°C and 0% RH.

^[d]^ To elucidate the proton transport mechanism, the activation energies were determined by applying linear regression (least‑squares fitting) to the Arrhenius plots^[29]^, with the results shown in Figure S20.

**Table S10**. Ohmic resistance, conductivity and fuel cells data of the assembled MEAs based on amine-anhydride crosslinked membranes

| **Category** | **Membrane ohmic resistance （Ω）** | **Conductivity （mS cm^-1^）** | **Limiting current density at 0.3 V (mA cm^-2^)** | **Power density**  **（mW cm^-2^）** |
| --- | --- | --- | --- | --- |
| TBAm-PBI-6F | 715 | 160.7 | 1167.6 | 350.3 |
| TBAm-PBI-NA | 520 | 199.4 | 1444.6 | 403.4 |
| TBAm-PBI-TB | 385 | 264.7 | 1487.2 | 446.2 |

**References:**

1. D. Van Der Spoel, E. Lindahl, B. Hess, G. Groenhof, A. E. Mark, and H. J. C. Berendsen, “GROMACS: Fast, flexible, and free,” *Journal of Computational Chemistry* 26 (2005): 1701. https://doi.org/10.1002/jcc.20291.

2. S. Pronk, S. Páll, R. Schulz, et al., “GROMACS 4.5: a high-throughput and highly parallel open source molecular simulation toolkit,” *Bioinformatics* 29 (2013): 845. https://doi.org/10.1093/bioinformatics/btt055.

3. M. J. Abraham, T. Murtola, R. Schulz, et al., “GROMACS: High performance molecular simulations through multi-level parallelism from laptops to supercomputers,” *SoftwareX* 1–2 (2015): 19. https://doi.org/10.1016/j.softx.2015.06.001.

4. H. J. C. Berendsen, D. van der Spoel, and R. van Drunen, “GROMACS: A message-passing parallel molecular dynamics implementation,” *Computer Physics Communications* 91 (1995): 43. https://doi.org/10.1016/0010-4655(95)00042-E.

5. L. Martínez, R. Andrade, E. G. Birgin, and J. M. Martínez, “PACKMOL: A package for building initial configurations for molecular dynamics simulations,” *Journal of Computational Chemistry* 30 (2009): 2157. https://doi.org/10.1002/jcc.21224.

6. W. L. Jorgensen, D. S. Maxwell, and J. Tirado-Rives, “Development and Testing of the OPLS All-Atom Force Field on Conformational Energetics and Properties of Organic Liquids,” *Journal of the American Chemical Society* 118 (1996): 11225. https://doi.org/10.1021/ja9621760.

7. T. Lu, and F. Chen, “Multiwfn: A multifunctional wavefunction analyzer,” *Journal of Computational Chemistry* 33 (2012): 580. https://doi.org/10.1002/jcc.22885.

8. Becke. A. D, “Density‐functional thermochemistry. III. The role of exact exchange,” *Journal of Chemical Physics* 98 (1993): 5648-5652. https://doi.org/10.1063/1.464913.

9. Lee. C, Yang. W, and Parr. R. G, “Development of the Colle-Salvetti correlation-energy formula into a functional of the electron density,” *Physical Review B* 37 (1988): 785-789. https://doi.org/10.1103/PhysRevB.37.785.

10. Grimme. S, Antony. J, Ehrlich. S, et al., “A consistent and accurate ab initio parametrization of density functional dispersion correction (DFT-D) for the 94 elements H-Pu,” *Journal of Chemical Physics* 132 (2010): 154104. https://doi.org/10.1063/1.3382344.

11. A. D.McNaught, and A. Wilkinson, *Compendium of Chemical Terminology*, 2nd edition Blackwell Scientific Publications, Oxford (1997).

12. Kenichi. Fukui, “The path of chemical reactions - the IRC approach,” *Accounts of Chemical Research* (1981). https://doi.org/10.1021/ar00072a001.

13. C. Pardo, I. Alkorta, and J. Elguero, “A DFT study of the geometric, magnetic NMR chemical shifts and optical rotation properties of Tröger’s bases,” *Tetrahedron: Asymmetry* 17 (2006): 191-198. https://doi.org/10.1016/j.tetasy.2005.12.013.

14. V. Sridharan, S. Saravanan, S. Muthusubramanian, and S. Sivasubramanian, “NMR investigation of hydrogen bonding and 1,3-tautomerism in 2-(2-hydroxy-5-substituted-aryl) benzimidazoles,” *Magnetic Resonance in Chemistry* 47 (2005): 551. https://doi.org/10.1002/mrc.1588.

15. J. Ji, H. Li, W. Wang, et al., “Silane-crosslinked polybenzimidazole with different hydroxyl content for high-temperature proton exchange membrane,” *Journal of Membrane Science* 694 (2024): 122423. https://doi.org/10.1016/j.memsci.2024.122423.

16. B. Zheng, C. Deng, R. Luo, S. Gao, F. Ji, and D. Wang, “Mechanically strengthened polybenzimidazole membrane via a two-step crosslinking strategy for high-temperature proton exchange membrane fuel cell,” *Journal of Power Sources* 603 (2024): 234369. https://doi.org/10.1016/j.jpowsour.2024.234369.

17. G. Liu, H. Pan, S. Zhao, Y. Wang, H. Tang, and H. Zhang, “Grafting of Amine End-Functionalized Side-Chain Polybenzimidazole Acid–Base Membrane with Enhanced Phosphoric Acid Retention Ability for High-Temperature Proton Exchange Membrane Fuel Cells,” *Molecules* 29 (2024): 340. https://doi.org/10.3390/molecules29020340.

18. A. Gao, W. Wang, P. Wang, et al., “Nitrogen‐Rich Rigid Polybenzimidazole With Phosphoric Acid Shows Promising Electrochemical Activity and Stability for High‐Temperature Proton Exchange Membrane Fuel Cells,” *Advanced Functional Materials* 33 (2023): 2305948. https://doi.org/10.1002/adfm.202305948.

19. J. Peng, X. Fu, J. Luo, L. Wang, and X. Peng, “Fabrication of high performance high-temperature proton exchange membranes through constructing stable cation-rich domain in polybenzimidazole membrane,” *Chemical Engineering Journal* 453 (2023): 139609. https://doi.org/10.1016/j.cej.2022.139609.

20. L. Li, L. Guo, L. Wang, Q. Wang, and J. Yang, “Covalently crosslinked poly(biphenyl dimethylamino benzene) membranes for high temperature proton exchange membrane fuel cells,” *Polymer* 300 (2024): 126992. https://doi.org/10.1016/j.polymer.2024.126992.

21. Y. Bai, D. Han, M. Xiao, et al., “New anhydrous proton exchange membranes based on polypyrrolone (PPy) for high-temperature polymer electrolyte fuel cells,” *Journal of Power Sources* 563 (2023): 232823. https://doi.org/10.1016/j.jpowsour.2023.232823.

22. A. Wu, J. Liu, J. Huang, et al., “Constructing High-Density Hydrogen Bonding Networks via Introducing the Bipyridine Group for High-Performance Fuel Cell Proton Exchange Membranes,” *ACS Applied Energy Materials* 5 (2022): 11815. https://doi.org/10.1021/acsaem.2c02346.

23. T. Wang, Y. Jin, T. Mu, T. Wang, and J. Yang, “Tröger's base polymer blended with poly(ether ketone cardo) for high temperature proton exchange membrane fuel cell applications,” *Journal of Membrane Science* 654 (2022): 120539. https://doi.org/10.1016/j.memsci.2022.120539.

24. J. Dai, Y. Zhang, C. Gong, Y. Wan, and Y. Zhuang, “Soluble polybenzimidazoles incorporating Tröger’s base for high-temperature proton exchange membrane fuel cells,” *Chemical Engineering Journal* 466 (2023): 143151. https://doi.org/10.1016/j.cej.2023.143151.

25. J. Dai, J. Zhong, J. Luo, et al., “Soluble Troger’s base-based polybenzimidazoles containing naphthalene units with improved phosphoric acid tolerance for use as high-temperature proton exchange membranes,” *Journal of Membrane Science* 700 (2024): 122657. https://doi.org/10.1016/j.memsci.2024.122657.

26. S. Li, and J. R. Fried, “Ab Initio Study of Proton Transfer and Interfacial Properties in Phosphoric Acid-Doped Polybenzimidazole,” *Macromolecular Theory and Simulations* 22 (2013): 410. https://doi.org/10.1002/mats.201300002.

27. S. Li, J. R. Fried, J. Sauer, J. Colebrook, and D. S. Dudis, “Computational chemistry and molecular simulations of phosphoric acid,” *International Journal of Quantum Chemistry* 111 (2011): 3212. https://doi.org/10.1002/qua.22702.

28. J.P. Melchior, G. Majer, and K.D. Kreuer, “Why do proton conducting polybenzimidazole phosphoric acid membranes perform well in high-temperature PEM fuel cells?,” *Physical Chemistry Chemical Physics* 19 (2017): 601. https://doi.org/10.1039/C6CP05331A.

29. J.N. Lu, S.F. Zhou, S.J. Zhang, C.X. Zhang, and Q.L. Wang, “ Remarkable Enhancement of Proton Conductivity by Introducing Imidazole into MOFs and Forming Composite Membranes,” *European Journal of Inorganic Chemistry* 6 (2019): 1434. https://doi.org/10.1002/ejic.201801084.
